# Dual role of ER stress in response to metabolic co-targeting and radiosensitivity in head and neck cancer cells

**DOI:** 10.1007/s00018-020-03704-7

**Published:** 2020-11-23

**Authors:** Oleg Chen, Friederike Manig, Loreen Lehmann, Nagwa Sorour, Steffen Löck, Zhanru Yu, Anna Dubrovska, Michael Baumann, Benedikt M. Kessler, Oleh Stasyk, Leoni A. Kunz-Schughart

**Affiliations:** 1 OncoRay, National Center for Radiation Research in Oncology, Faculty of Medicine and University Hospital Carl Gustav Carus, TU Dresden and Helmholtz-Zentrum Dresden-Rossendorf, Fetscherstraße 74, 01307 Dresden, Germany; 2 Department of Cell Signaling, Institute of Cell Biology, National Academy of Sciences of Ukraine, Lviv, Ukraine; 3 Chair of Food Chemistry, TU Dresden, Dresden, Germany; 4 German Cancer Consortium (DKTK), Partner Site Dresden, Germany; 5 German Cancer Research Center (DKFZ), Heidelberg, Germany; 6 Department of Radiation Oncology, University Hospital Carl Gustav Carus, Dresden, Germany; 7 Target Discovery Institute, Nuffield Department of Medicine, University of Oxford, Oxford, UK; 8 Institute of Radiooncology, Helmholtz-Zentrum Dresden-Rossendorf, Dresden, Germany; 9 National Center for Tumor Diseases (NCT), Partner Site Dresden, Germany

**Keywords:** Metabolic targeting, Arginine-deprivation therapy, ER stress, Canavanine, Radiosensitization, Head and neck squamous carcinoma, 3-D culture

## Abstract

Arginine deprivation therapy (ADT) is a new metabolic targeting approach with high therapeutic potential for various solid cancers. Combination of ADT with low doses of the natural arginine analog canavanine effectively sensitizes malignant cells to irradiation. However, the molecular mechanisms determining the sensitivity of intrinsically non-auxotrophic cancers to arginine deficiency are still poorly understood. We here show for the first time that arginine deficiency is accompanied by global metabolic changes and protein/membrane breakdown, and results in the induction of specific, more or less pronounced (severe vs. mild) ER stress responses in head and neck squamous cell carcinoma (HNSCC) cells that differ in their intrinsic ADT sensitivity. Combination of ADT with canavanine triggered catastrophic ER stress via the eIF2α-ATF4(GADD34)-CHOP pathway, thereby inducing apoptosis; the same signaling arm was irrelevant in ADT-related radiosensitization. The particular strong supra-additive effect of ADT, canavanine and irradiation in both intrinsically more and less sensitive cancer cells supports the rational of ER stress pathways as novel target for improving multi-modal metabolic anti-cancer therapy.

**Electronic supplementary material:**

The online version of this article (10.1007/s00018-020-03704-7) contains supplementary material, which is available to authorized users.

## Introduction

During the past decade, significant progress has been accomplished in the development of enzymotherapeutic arginine deprivation therapy (ADT) for anticancer treatment. Systemic withdrawal of arginine (Arg) from the patients’ blood stream can be achieved using one of two types of Arg-degrading enzymes, recombinant human arginase (rhARG) or bacterial arginine deiminase (ADI). Both enzymes are available in pegylated form and have entered pre-clinical and clinical phase I/II trials, in particular for the treatment of putative Arg-auxotrophic tumors [1–6]. The proposed cancer cells targeted by ADT are incapable of Arg de novo synthesis mainly due to deficiency in argininosuccinate synthetase (ASS), the rate-limiting enzyme of citrulline (Cit) to Arg conversion [3, 7, 8].

Recently, we demonstrated a strong synergistic effect when ADT was combined with low concentrations of Cav [9–13]. The natural Arg analog Canavanine (Cav) can effectively compete with Arg for arginyl tRNA synthase and incorporates into cellular proteins, thereby causing conformational changes and impairing enzymatic activities and functions [14]. ADT was also found to efficiently sensitize even non-auxotrophic colorectal cancer and glioblastoma cells to irradiation [10, 13, 15]. However, the molecular mechanism(s) underlying the cancer cells’ reprogramming to Arg deficiency and its link to radiosensitization are virtually unknown.

Our previous studies indicate that cancer cells in the absence of exogenous Arg rapidly deplete their internal Arg pools, repress global protein translation by activation of the GCN2-mediated pathway, and inhibit mTOR signaling in both 2-D and 3-D models [16]. Prolonged ADT was also shown to induce endoplasmic reticulum (ER) stress responses [11]. Activated ER stress pathways primarily have pro-survival functions to resolve and overcome the stress conditions. In case of massive or unresolved ER stress, the pathways switch to cause apoptotic cell death [17]. It remains unclear, how and when this switch is flicked in cancer cells under Arg-deprived conditions in mono- and combinatorial treatment settings, and whether interference with selective ER stress responses could further improve ADT outcome.

In the present study, we for the first time examined the mechanistic role of ER stress in radiosensitization and apoptosis induction upon mono-ADT (Arg deprivation mono-therapy) and comb-ADT (Arg deprivation therapy combined with Cav) in head and neck squamous cell carcinoma (HNSCC) models. HNSCC comprise a heterogeneous group of cancer subtypes accounting for > 750,000 cases and ~ 350,000 disease-related deaths worldwide each year [18]. A minor subgroup of HNSCC causally relates to HPV infection; these HPV^+^ HNSCC show a specific molecular pathogenesis with favorable prognosis and were intentionally excluded from our investigation. Most HNSCC patients (~ 70%) are first diagnosed with large primary cancers and/or at locoregionally advanced stages. The standard-of-care treatment in those cases is multimodal and includes radio(chemo)therapy. However, the survival outcome of the patients remains modest (5-year survival 25–49% depending on location). Combining mono-ADT or comb-ADT with ionizing radiation could thus be a promising alternative, individualized treatment strategy. With a systematic study design, we identified responder and non-responder HNSCC models and found a dualistic role of selectively activated ER stress pathways in cell death and radiosensitization. New fundamental insight in the role of triggered ER stress response upon mono- and comb-ADT is given.

## Materials and methods

### HNSCC cell lines

Nine established human HNSCC lines were used in this study. The origin of the cell lines in our laboratory has been highlighted earlier in [19, 20]. In brief, UT-SCC-5, UT-SCC-8, UT-SCC-14 and UT-SCC-15 cells were kindly provided by Prof. Reidar Grenman MD PhD, from the University of Turku (Finland); HSC-4 and SAS cells were obtained from the HSRRB (Japan), XF354 cells from the German Cancer Research Center (DKFZ, Germany), and Cal-33 cells from the German Collection of Microorganisms and Cell Cultures (DSMZ, Germany). The FaDu cells applied herein represent a subline of FaDu-ATCC HTB-43 as pointed out earlier [21]. All cell lines were routinely tested free of mycoplasms using a PCR Mycoplasma Kit (AppliChem). Authentication and purity of the HNSCC cell line panel were verified prior to use via multiplex PCR kits, i.e. Mentype^®^ Nonaplex^QS^ Twin (Biotype) and the PowerPlex^®^ 16 System (Promega) at the Institute of Legal Medicine (TU Dresden, Germany). Cultures were routinely grown from the validated frozen stocks for ≥ 2 to ≤ 20 passages (< 120 CPD—cumulative population doublings).

### Monolayer and spheroid routine culturing

All cells were cultured as monolayers in standard Dulbecco’s Modified Eagle Medium (DMEM) with l-glutamine, d-glucose (1 g/L) and 25 mM HEPES supplemented with 10% heat-inactivated fetal calf serum (FCS) and 1% penicillin/streptomycin (10,000 U/mL /10 mg/mL). Exponentially growing cultures were kept at 37 °C in a humidified atmosphere with 8% CO_2_ in air. Exponential monolayer cultures were enzymatically dissociated using a solution of 0.05% trypsin and 0.02% in phosphate-buffered saline (PBS) to obtain single cell suspensions for passaging and experimental setup. All media, media supplements, solutions and buffers for both routine culturing and therapeutic intervention were from PAN-Biotech if not stated otherwise. A CASY^®^ TTC device (Roche Innovatis) was applied for cell counting, cell volume analysis and culture quality assessment.

Spheroids were cultured in conventional liquid overlay as described previously [22]. In brief, 1000 SAS cells or 2500–3500 FaDu cells derived from exponentially growing monolayer cultures were seeded in 200 µL of regular DMEM per well in 96-well plates coated with 1.5% agarose (Sigma-Aldrich). After 4 days of initiation, all spheroids should have reached a standard diameter of 370–400 µm for both cell lines. Spheroids were fed every 48–72 h by 50% medium renewal independent of the type of treatment. Spheroid integrity and growth kinetics were routinely monitored by semi-automated measurement of spheroid diameters (and volumes) from phase contrast images taken with an adequately equipped Axiovert200M microscope (Carl Zeiss MicroImaging) as detailed in [15, 22].

### Therapy implementation: mono-ADT, comb-ADT, irradiation

Mono-ADT in culture was mimicked in vitro using formulated Arg-free DMEM. The appropriate control medium was generated by supplementation of Arg-free DMEM with Arg to a final concentration according to standard DMEM. Media were supplemented with 10% dialysed FCS, depleted of molecules below 10 kDa (i.e. amino acids). For practical reasons, mono-ADT was achieved for selected experimental series (i.e. metabolomics study) by exposure to 2 U/mL yeast-derived recombinant human arginase I (rhARG; provided by the Institute of Cell Biology, NASU, Lviv, Ukraine) in the presence of physiological Cit according to a previous study [15, 16]. Successful deprivation of intracellular Arg was monitored by high-resolution mass spectrometry as described later (Fig. S5A).

Comb-ADT in culture was achieved by formulated Arg-free DMEM supplemented with 0.01–0.1 mM of the natural arginine analog Cav (Sigma-Aldrich). In some experiments, the precursor of Arg–Cit was added at a physiological concentration of 0.04 mM. ER stress modulators such as 0.02 mM salubrinal (Sal; Sigma-Aldrich) or 2% dimethyl sulfoxide (DMSO; Sigma-Aldrich) were applied 1 h before and throughout exposure to mono- or comb-ADT. A 4 mM stock solution of Sal in DMSO was prepared, stored at − 20 °C and always freshly diluted in medium prior to use; accordingly, control cells were exposed to medium containing 0.5% of DMSO.

Irradiation was performed at room temperature with an X-ray approach (200 kV, 0.5 mm Cu filter, YxlonY.TU 320, Yxlon.international) and single doses of 0–12 Gy for 2-D and 0–30 Gy for 3-D cultures (dose rate ~ 1.3 Gy/min).

### 2-D and 3-D growth and re-growth experiments

Monolayer cells were seeded at a density of 1 × 10^4^ cells per well in 6-well culture dishes. Cells were allowed to attach overnight, washed with PBS, and then medium was exchanged to provide an Arg-free environment. At defined time intervals, cell numbers in at least three wells were measured using the CASY^®^ TTC analyzer after cell dissociation. To evaluate growth restoration capability upon ADT, Arg-free medium was exchanged in a sufficient number of wells by standard DMEM containing 0.4 mM Arg and membrane-intact cells were counted 3 days later.

Spheroids with a diameter of 370–400 µm were obtained as described above, collected, carefully washed with PBS and individually transferred into new agarose-coated 96-well plates prepared from serum-free, Arg-free medium for controlled exposure to Arg-free conditions with or without 0.04 mM Cit and 0.1 mM Cav (Sigma-Aldrich). After defined treatment intervals, spheroids were re-transferred into standard supplemented DMEM and monitored for at least 10–15 more days. A minimum of 30 spheroids were analyzed per treatment arm. Growth delay *t*_SGD_ represents the different time intervals of treated versus control spheroids to reach 5-times the starting volume (5 × *V*_0_), with the starting volume (*V*_0_) being defined as the spheroid volume directly before the onset of treatment, respectively. To determine spheroid growth delay, the time of each spheroid to reach 5 × *V*_0_ was evaluated. For this purpose, the monitoring time points closest to the individual 5 × *V*_0_ were identified, and regression analyses through the logarithm of the corresponding spheroid volumes were used to estimate the individual time to reach 5 × *V*_0_. The *t*_SGD_ values were then calculated by subtracting the average time of control spheroids to reach their 5 × *V*_0_ from the individual time periods required by the treated spheroids and are documented as means ± SD.

### 2-D clonogenic survival assay

Exponentially growing HNSCC cells were dissociated, single-cell suspensions were prepared in standard DMEM and seeded cell line-dependent at low (between 100 and 10,000) in 6-well plates to allow counting of single-cell colonies. After overnight incubation to allow cell adherence, supernatants were removed, cells were carefully washed with PBS and specific media for mono- or comb-ADT were added (2 mL/well) as described in “Therapy Implementation”. Single-dose irradiation was performed after 24 h of treatment followed by medium exchange to restore Arg-rich conditions for a period of ≥ 5–6 cell divisions estimated from the cell line-specific doubling times of the respective controls. Hence, the culturing time ranged between 8 and 14 days for the cell line panel except for UT-SCC-8 cells which required a period of 20 days due to their low growth rate under the same conditions. An additional, careful washing step with PBS was performed after irradiation in experimental series with comb-ADT in the absence and presence of ER stress modulators. Control cells and wells, respectively, under + Arg conditions were also irradiated after attachment to exclude effects due to cell divisions before irradiation. After the defined, cell-line specific growth intervals, cells were fixed, stained and colonies with > 50 cells were manually counted at low magnification to calculate plating efficiencies (PE) and survival fractions (SF) relative to controls. Cell survival curves were fitted by employing the linear-quadratic model as described in [15].

### Spheroid control probability assay

Spheroids with standard size were exposed to mono-ADT or comb-ADT for 1 day as described in “Therapy implementation", and were then irradiated with 0–30 Gy single-dose X-ray. Subsequently, treatment was terminated by exchanging and supplementing the Arg-free media to restore Arg-rich conditions. Control spheroids were irradiated at day 4 in culture to minimize growth effects, i.e. spheroids under mono- and comb-ADT did not grow and showed the same size and volume range on day 5 before irradiation as control spheroids in standard DMEM at day 4 in culture. Spheroid feeding after treatment over the entire post-treatment monitoring time of 60 days was carried out with standard DMEM. Spheroids which did not enlarge over at least three consecutive time points and did not regrow to > 200 µm were declared as "controlled".

Growth delay values (*t*_SGD_) as depicted in “Growth and regrowth experiments” could be reliably calculated only for treatment arms showing 100% regrowth, e.g. for low doses of irradiation. For higher doses, the proportion of controlled spheroids was documented as function of time. The spheroid control probability (SCP) refers to the proportion of spheroids that has lost regrowth capacity upon treatment. SCP values were documented as function of the irradiation dose, and curves were fitted with a sigmoid dose–response model according to the tumor control probability in vivo assay (e.g. [19].) using the following function (*D*—dose; *a*, *b*—variables)

$${\text{SCP}} = 1 - \frac{1}{{1 + {\text{e}}^{( - a - b\ln D)} }}.$$

Subsequently, the spheroid control dose 50 (SCD_50_–radiation dose leading to a loss of growth recovery in 50% of the spheroids) was calculated, and the quotient of SCD_50_ values for treatment arms of interest was determined as dose reduction factors (DRF).

### Reverse transcription (RT)-PCR analysis

Cells were seeded at defined concentration in monolayer culture and samples were collected at different time points (0–120 h) throughout treatment to isolate total cellular RNA using the RNeasy Mini Kit (Qiagen) according to the manufacturer’s instructions. RNA concentrations and purity were assessed spectrophotometrically with a Nanodrop 1000 spectrophotometer (Thermo Fisher Scientific). cDNA was synthesized from each sample using the Verso cDNA Synthesis Kit (Thermo Fisher Scientific) and 1 µg of total RNA. PCR was carried out in an MJ Research PTC-200 Thermal Cycler (Bio-Rad) using the GoTaq Flexi DNA Polymerase Kit (Promega) and specific primers for relevant ER stress response genes. Primer pairs with product sizes are listed in Table S1. Conditions for the PCRs were as follows: initial denaturation at 95 °C for 7 min, denaturation in cycles at 95 °C for 30 s, annealing 46–55 °C for 30 s, extension 72 °C for 1 min, and a final step at 72 °C for 5 min. PCR products were separated by 2.0% agarose gel electrophoresis, visualized by RedSafe (iNtRON Biotechnology) dye staining, and documented using the GeneGenius Gel Imaging System (Syngene). The spliced (415 bp) and unspliced (441 bp) variants of XBP1 mRNA were resolved by 4% agarose gel electrophoresis as described previously [23]. Human-specific *ACTB* gene primers were applied as reference.

### Real-time quantitative (q-)PCR analysis

Total cellular RNA from defined treated and untreated monolayer cell samples was extracted and cDNA was synthesized as described above. The specific primer sequences for the analyzed genes were identical to the RT-PCR approaches (Table S1). cDNA was diluted (1:10) and the one step PCR reaction was performed in a total volume of 20 µL using the GoTaq qPCR Master Mix (Promega) according to the manufacturer’s protocol. The PCR condition was set at an initial denaturation at 95 °C for 10 min, 45 cycles at 95 °C for 15 s and 57 °C for 1 min. Data were collected and analyzed using the Applied Biosystems StepOnePlus Real-Time PCR System with v.2.2.2 StepOne Software (Life Technologies, Applied Biosystems). The relative gene expression of control versus treated cells was assessed by the comparative threshold cycle (ΔΔCt) method using *ACTB* as reference control; values ≥ twofold were considered as differentially regulated.

### siRNA transfection

Small interfering (si)RNAs for *IRE1, GADD34, ATF3, ATF4* and *CHOP* were purchased from Eurofins Genomics. Their targeting sequences are given in Table S2. Cells transfected with unspecific siRNAs (scrambled siRNA #1, #2 and #3) were used as negative control. Transfection was conducted with the Lipofectamine™ RNAiMAX (Invitrogen) according to recommendations of the manufacturer. 48 h after transfection, cells were treated with mono- or comb-ADT for defined time intervals and then processed for PCR, western blotting or clonogenic survival assay.

### Western blotting (WB)

Western blots were performed using whole cell protein extracts from 2-D and 3-D cultures as described previously [16]. Various primary antibodies (listed in Table S3) were applied according to the manufacturers’ instructions to monitor specific non-phosphorylated and phosphorylated proteins of interest. Horseradish peroxidase-conjugated goat anti-mouse or anti-rabbit IgG secondary antibodies (Dako) were used for detection, and the immuno-reactive bands were visualized using a chemiluminescence detection kit (Santa Cruz Biotechnology) followed by documentation on X-ray films (GE Healthcare). Western blots were performed for *N* = 3 independent experiments and band intensities were assessed in 2–3 densitometric scans per experiment (technical replicates) using the ImageJ software. Protein signals were normalized to α-tubulin or GAPDH loading controls and related to an internal standard protein sample, e.g. scrambled siRNA-transfected cells.

### Metabolomics

Large numbers of FaDu and SAS spheroids (400–500 of each) were prepared at standard size (see above) and then exposed to control or mono-ADT conditions for 24 h. Three aliquots of six spheroids were taken per treatment arm to determine the average cell number per spheroid in the differently treated spheroid populations; FaDu and SAS spheroids were enzymatically dissociated according to an established protocol [24], and cells were counted using a Scepter Cell Counter Sensor (Millipore). Another 180 non-dissociated spheroids were then pooled per treatment arm, transferred into 50 mL Falcon tubes, washed twice with 30–50 mL ice-cold PBS including short spins (500 g) at 4 °C and finally resuspended in 500 µL ice-cold 50% methanol for transfer into low-binding 1.5 mL Eppendorf tubes. Samples were frozen immediately and stored at − 80 °C until analysis. Metabolites were extracted as described earlier [25].

Samples obtained from two independent experiments with SAS and FaDu spheroids were then analyzed using two different metabolic profiling platforms. The first was a two-dimensional gas chromatography workflow combined with high-speed scan single quadrupole mass spectrometry (GCxGC-qMS) as detailed in [25]. The second analysis platform was a liquid chromatography–mass spectrometry (LC–MS) system consisting of an Agilent 1290 Infinity ultra-high-performance liquid chromatography system (UHPLC) equipped with a quaternary pump delivery system (G4204A), a HiP autosampler (G4226A), a column thermostat (G1316C). A BEH C18 XP Column, (130 Å, 1.7 µm, 2.1 mm × 150 mm; Waters) was used for untargeted metabolomics profiling. Alternatively, an ACQUITY Glycoprotein Amide Column, (300 Å, 1.7 µm, 2.1 mm × 150 mm; Waters) was used for arginine analysis. The UHPLC system was coupled to a 6560 Ion mobility QTOF LC/MS mass spectrometer (Agilent Technologies) equipped with a Jetstream ESI-AJS source.

For arginine analysis by LC–MS, dried metabolite extracts were re-suspended 20 μL per million cells in water (LC–MS graded from Merck) with 0.1% formic acid (FA, LC–MS graded from Fisher Scientific). Data were acquired by LC–MS in QToF mode using positive electrospray ionisation (ESI+). Two reference ions, *m/z* 121.0508 and 922.0097 were used as internal standards. The Dual AJS ESI settings were as follows: gas temperature: 300 °C, the drying gas: 8 L/min, nebulizer 35 MPa, sheath gas temperature 350 °C, sheath gas flow 11 L/min, Vcap 3.500 V and nozzle voltage 1000 V. The fragmentor of the mass spectrometer TOF was set to 400 V.

The UHPLC gradient was composed of 20% buffer A [water (0.1% FA)] and 80% buffer B [Acetonitrile (LC–MS graded from Merck) (0.1% FA)] with a flow rate of 0.30 mL; 0–8 min 20–35% A; 8–9 min 35–20% A. The gradient was followed by a 3-min post-time to re-equilibrate the column. The external standard, l-arginine monohydrochloride (Sigma-Aldrich), was used for its identification.

For untargeted metabolomics analysis by LC–MS, samples of metabolite extracts were re-suspended in 20 μL per million cells of 60% acetonitrile in water (5 mM ammonium formate [LC graded from Sigma-Aldrich)]. The data were acquired by LC–MS in QToF mode using positive electrospray ionisation (ESI+). Two reference ions, *m/z* 121.0508 and 922.0097 were used as internal standards. The Dual AJS ESI settings were as follows: gas temperature: 325 °C, the drying gas: 5 L/min, nebulizer 35 MPa, sheath gas temperature 275 °C, sheath gas flow 12 L/min, Vcap 4.000 V and nozzle voltage 500 V. The fragmentor of the mass spectrometer TOF was set to 400 V.

The gradient in a total analysis time of 42 min started by a 5-min isocratic gradient composed with 95% buffer A [60% acetonitrile in water (5 mM ammonium formate)] and 5% buffer B [10% acetonitrile in 2-propanol (LC–MS graded from Fisher Scientific) (5 mM ammonium formate)] with a flow rate of 0.25 mL and was followed by steps: 5–7 min 95–50% A; 7–25 min 50–5% A; 25–30 min 5% A; 30–30.1 min 5–95% A 30.1–35 min 95% A. The gradient was followed by a 7 min post time to re-equilibrate the column.

The raw LC–MS data were processed and analyzed using the MassHunter Workstation software package (Agilent Technologies, version B8.0). Further data analysis was performed through XCMS Online [26, 27]. GCxGC data analysis was performed as described previously [25].

### Experimental setup with non-malignant cells

The human retinal pigment epithelial cell line ARPE-19 (ATCC), human foreskin fibroblasts established earlier in our laboratory [28] and human umbilical vein endothelial cells (HUVEC) isolated at the Division of Vascular Endothelium and Microcirculation, Department of Medicine III, Medical Faculty and University Hospital Carl Gustav Carus, TU Dresden, Germany, according to [29] served as normal cell reference. In contrast to the established cell lines, HUVEC and fibroblasts were re-cultured from low passage frozen stocks (P1 and P3, respectively), and used for 3-D cell culture experiments at with a CPD < 10. Standard DMEM as described for cell lines was also used for culturing normal cells with the following exception: all media for HUVEC were additionally supplemented with 0.5% self-isolated retina calf eye growth supplement as highlighted in [29]. One batch of fibroblasts (VF2) and two different pooled HUVEC preparations were applied in the present study. VF2 fibroblast and ARPE-19 cells were studied in *N* ≥ 3 independent experiments with *n* = 2–3 biological repeats while the HUVEC preparations were used in individual experimental series with *n* = 3 biological repeats.

For experimental setup, normal cells were seeded into 96-well plates according to the spheroid liquid overlay approach; 1 × 10^4^ (HUVEC, fibroblasts) or 5000 cells (ARPE-19) were inoculated per well and the resulting 3-D cultures (single aggregate per well) at day 4 were monitored and treated with mono- or comb-ADT for up to 7 days. Two to three aliquots of 8–12 spheroids from each treatment arm were collected at defined time points and enzymatically dissociated for cell counting. The average number of membrane-intact cells per spheroid was determined for each treatment arm and experiment, and the viability was assessed as cell number in treated relative to the respective untreated cultures that were kept under + Arg conditions for the same time period.

### Bioinformatics and statistical analyses

Bioinformatic analysis of the TCGA dataset was conducted on processed RNA-seq array, gene alteration data, and paired clinical feature data downloaded through the cBioPortal (http://www.cbioportal.org). Microarray data processing, Kaplan–Meier curves of patient data, heatmaps, correlation analyses and log rank statistics were performed in Small Utilities of Microarray Data Analysis (SUMO) software.

All values within the text and figures referring to 2-D culture data are presented as mean ± SD from three independent experiments each with ≥ 3 biological replicates (*N* = 3, *n* ≥ 3), as described in the figure legends. Treatment groups of interest were compared for all time points using a two-way ANOVA test. Clonogenic survival was characterized with the linear-quadratic model and *p *values were determined by Mann–Whitney test with Bonferroni–Holm correction using IBM SPSS Statistics as described previously in [13, 15]. ANOVA comprising Bonferroni correction was applied for 3-D cell growth assays while logistic regression was employed to compare SCD_50_ values as well as DRF and to fit the corresponding spheroid control probability curves. Subsequent bootstrapping with 1000 samples was performed using STATA/SE 11.2 (StataCorp LLC, TX, USA) to determine 95% confidence intervals and estimate statistical significance. A minimum of 30 individual spheroids were monitored for each treatment arm and irradiation dose. Values are documented according to their significance level as *p* < 0.05 (*), *p* < 0.01 (**), and *p* < 0.001 (***).

## Results

### Mono-ADT induces heterogeneous growth arrest in HNSCC cells

**Fig. 1:**
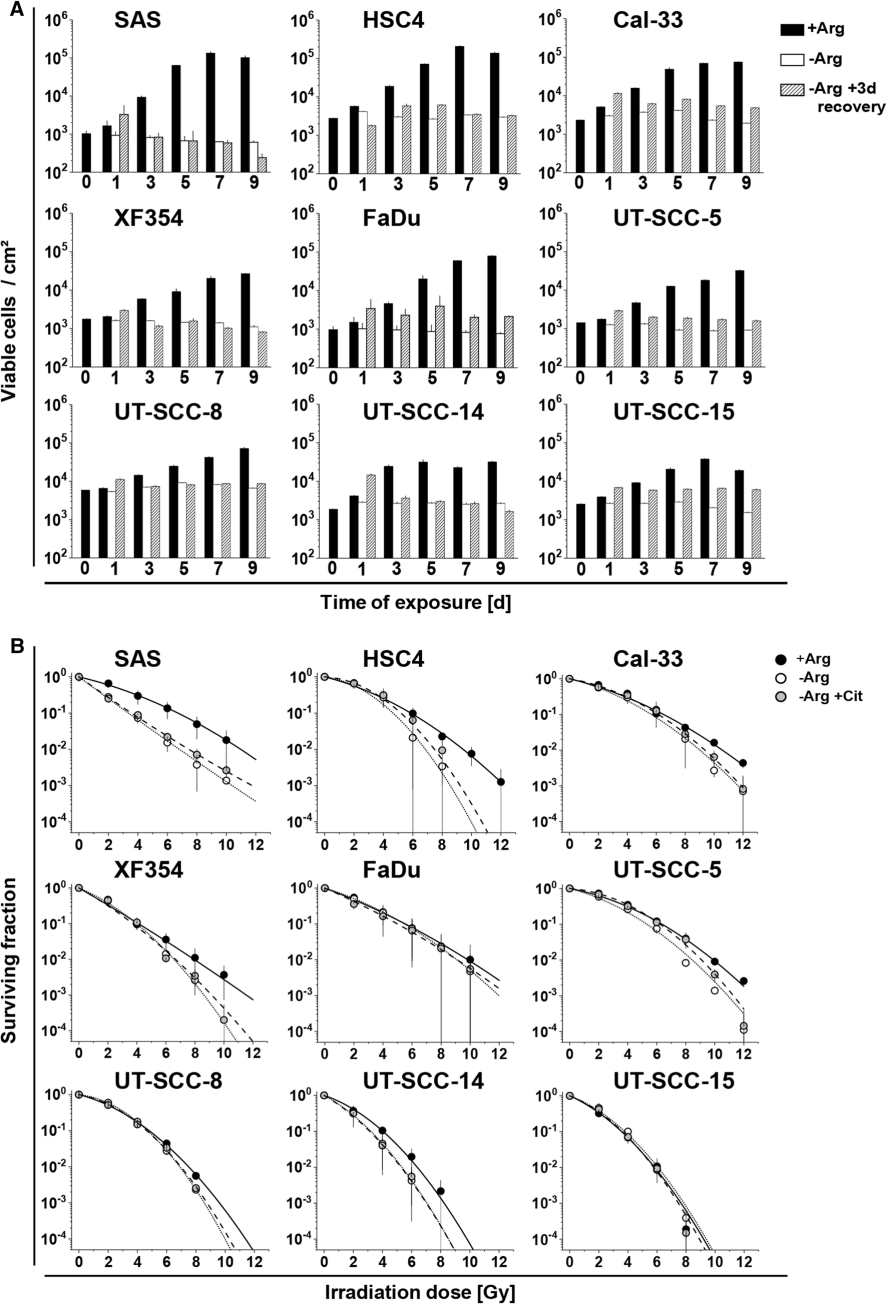
Mono-ADT induces heterogeneous growth arrest and efficiently sensitizes selected HNSCC cells to irradiation. All data show means (± SD) from three independent experiments of nine HNSCC cell lines in 2-D culture each with ≥ 3 biological replicates (N = 3, *n* ≥ 3). **a** Growth and regrowth of cells exposed to Arg-rich (+ Arg) or Arg-free conditions (− Arg) for 1–9 days. Medium was exchanged after treatment to standard (+ Arg) for an additional period of 3 days to allow growth recovery. **b** Clonogenic survival of cells pre-exposed for 24 h to + Arg or − Arg conditions with or without 0.04 mM Cit. Cells were irradiated with single doses of 0–12 Gy and then allowed to form single cell colonies. Data were fitted with a linear-quadratic model

To examine the efficacy of mono-ADT in HNSCC, we initially compared cell growth and re-growth capacity in 9 different HPV^−^ HNSCC cell lines in 2-D culture under control (+Arg) and Arg-free (−Arg) conditions over a period of up to 9 days. All cell lines responded to mono-ADT with an acute growth arrest (Fig. 1a; Table S4A), and in some cases the number of viable cells even decreased with exposure time indicating that Arg is an essential amino acid for HNSCC cell growth in vitro. Growth recovery (i.e. ability to resume growth) after different intervals of mono-ADT was assessed 3 days after termination of treatment and re-transfer to standard Arg-rich conditions. HNSCC cells showed a highly variable extend of growth restoration. The ability to recover after Arg starvation decreased time-dependently for SAS, HSC4, XF354, UT-SCC-8 and UT-SCC-14 cells, but remained largely unaltered after incremental periods of treatment for Cal-33, FaDu, UT-SCC-5, and UT-SCC-15 cells. This suggests that the latter cell lines are intrinsically more resistant (non-responders) to mono-ADT in spite of the general growth arrest during treatment (Fig. 1a; Table S4A).

It is well established that ASS is the rate-limiting enzyme for the biosynthesis of Arg from Cit and one determinant of Arg auxotrophic response in vivo [7, 8]. We, therefore, analyzed the levels of this marker enzyme in the HNSCC cell lines and, also monitored growth and growth recovery, respectively, upon exposure to Arg-deprived, Cit-conditioned (0.04 mM) medium, which better mimics the in vivo conditions of ADT. All HNSCC cells were ASS-positive. However, the heterogeneous ASS protein levels strongly correlating with *ASS1* gene expression (Fig. S1A–C) in the cell lines did not reflect their divergent capacity for sustained growth under mono-ADT in the presence of Cit (Fig. S1D). Although Cit in principle supported growth recovery after completion of the treatment, it could not rescue all sensitive cell lines from acute ADT-induced growth arrest.

### Mono-ADT efficiently sensitizes selected HNSCC cells to irradiation

Radio(chemo)therapy is the contemporary standard-of-care in the curative-intent management of HNSCC patients [30]. We, therefore, addressed the impact of mono-ADT (24 h pre-treatment) on the radioresponse of HNSCC cells via 2-D clonogenic survival assays. Significant radiosensitization upon mono-ADT was seen in six out of nine tested cell lines. No general overlap between mono-ADT dependent growth restoration capacity and radiosensitization was observed. Radiosensitization was particularly prominent in the intrinsic ADT-responder cell lines SAS, HSC4, and XF354 where physiological Cit concentrations turned out to be insufficient for compensating the effect (Fig. 1b; Table S4B), whereas FaDu, UT-SCC-5 and UT-SCC-15 proved to be general non-responders.

In contrast to the regrowth capacity, we observed a positive correlation of intermediate strength (not yet significant) between ASS protein content in the HNSCC models in vitro and their radiosensitivity as reflected by the dose required to reduce clonogenic survival to 0.1% (0.1% surviving fraction dose—SFD) (Fig. S2A). Regardless of pathophysiological phenomena that are not reflected in the in vitro assay, but can drive radioresistance in vivo such as hypoxia, the 0.1% SFD values well correlate with the in vivo “curative” endpoint TCD_50_ (irradiation dose required to control the disease in 50% of tumor-bearing mice) published earlier for xenografts derived from the same HNSCC cell line panel (Fig. S2B) [20]. Based on these data, we propose a link between ASS expression and intrinsic radioresistance in HNSCC cells which is apparently unrelated to the mono-ADT-induced radiosensitization mechanisms.

### Mono-ADT triggers massive and durable ER stress

Nutrient deficiency and cellular metabolic stress are sensed by the ER. Hence, mono-ADT most likely induces ER stress responses in HNSCC cells. We examined the activation of ER stress signaling cascades upon mono-ADT in two representative HNSCC cell lines, i.e. SAS as a responder and FaDu as a non-responder model. Levels of several marker genes and proteins involved in the ER stress response pathways were examined by RT-PCR and Western blot (WB) analyses. Notably, both HNSCC cell lines are defective in functional p53 (https://p53.iarc.fr/CellLines.aspx), and differential ADT-induced effects as documented in the following chapters can thus not be attributed to this tumor suppressor as indicated in earlier studies [15, 16].

**Fig. 2:**
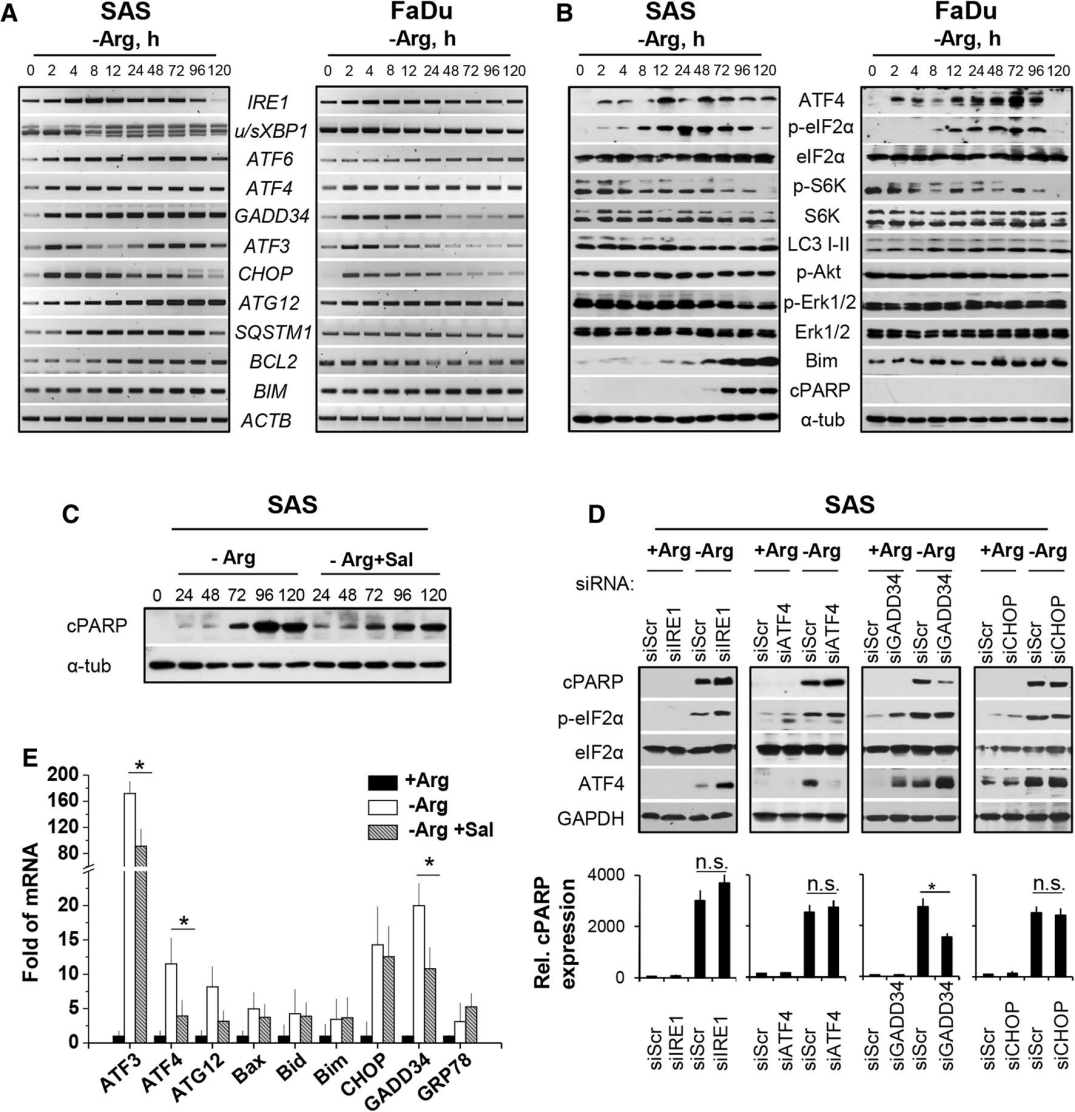
Mono-ADT triggers massive and durable ER stress which is partially linked to apoptosis. All data show representative or averaged results of three independent experiments (*N* = 3) for SAS (**a**–**e**) and FaDu (**a**/**b**) 2-D cultures; the ER stress modulator salubrinal (Sal) was added at 0.02 mM 1 h before ADT. **a** Representative RT-PCR data sets documenting the expression of several ER stress response genes in cells exposed to − Arg medium for up to 120 h or left untreated (0 h). *ACTB* gene was used as reference. **b** Representative western blots for various proteins of interest in cells treated according to **a**. α-tubulin (α-tub) was used as loading control. **c** Representative western blots of cPARP protein in SAS cells after different periods of mono-ADT (1–5 days) with or without Sal. **d** Representative western blots for proteins of interest in siRNA-mediated knockdown SAS cells treated with mono-ADT. Cells were transfected with siRNAs (*IRE1, ATF4, GADD34* and *CHOP*) and then exposed to + Arg or − Arg conditions for 72 h. Non-specific siRNAs were used as control (siScr). Mean values (± SD) from densitometric analysis of the relative cPARP expression normalized to GAPDH are documented in the lower panel; *p ≤ 0.05; *n.s. *not statistically significant. **e** Level of ER stress response genes measured by qPCR in SAS cells upon 72 h of Arg deprivation with or without Sal. Data normalized to *ACTB* gene are shown as means (± SD); *p ≤ 0.05

**Fig. 3:**
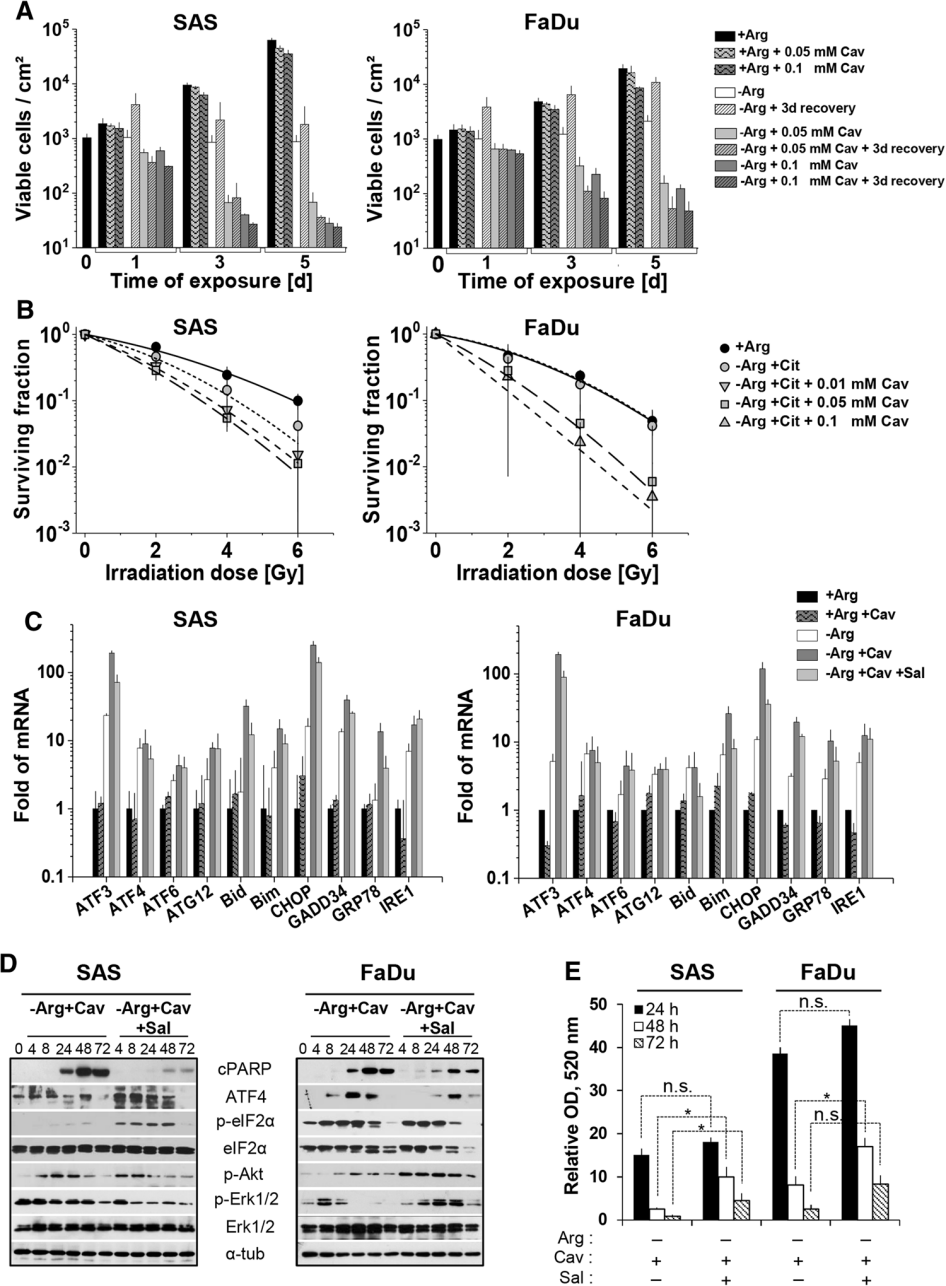
Arg analog–Cav strongly augments the efficacy of mono-ADT and results in catastrophic ER stress and apoptosis. All data show means (± SD) from three independent experiments with ≥ 3 biological replicates each (*N* = 3, *n* ≥ 3) in SAS and FaDu 2-D cultures; salubrinal (Sal) was applied according to Fig. 2. **a** Cell count per surface area after different periods of mono- and comb-ADT with 0.05–0.1 mM Cav (0–5 days) in the presence of 0.04 mM Cit and 3 days after termination of treatment (3-day recovery). **b** Clonogenic survival of cells irradiated with single doses of 0–6 Gy under either standard conditions or upon 24 h pre-exposure to − Arg medium with and without 0.04 mM Cit and 0.01–0.1 mM Cav. Data were fitted according to Fig. 1b. **c** Level of ER stress response genes of interest (qPCR) upon 24 h of Arg deprivation with and without 0.1 mM Cav and/or Sal. Data were normalized to *ACTB* mRNA level. **d** Representative western blots showing proteins of interest in cells incubated in − Arg medium with 0.1 mM Cav for up to 72 h with and without Sal; α-tubulin (α-tub) was used as loading control. **e** MTT assay readout reflecting the relative number of viable cells after 24–72 h comb-ADT with and without Sal. Data are presented as mean OD_520nm_ ± SD; *p ≤ 0.05; *n.s.* not statistically significant

Mono-ADT (in contrast to comb-ADT as documented later in Figs. 2e and 3c) did not significantly enhance the expression of the classical ER stress marker gene *GRP78* in the HNSCC cell line models. However, we detected a reproducible time-dependent upregulation of the ER sensor genes *IRE1* and *ATF6*, which was clearly more pronounced in SAS than FaDu cells (Fig. 2a). The *IRE1* mRNA level reached a maximum after 8–16 h of Arg deprivation and then gradually declined. Activated IRE1α cleaves its pre-mRNA substrate X-box binding protein 1 (*XBP1*) mRNA to initiate an unconventional splicing reaction and produce mature *sXBP1* (spliced *XBP1*) mRNA [23]. A strong splicing reaction of *XBP1* mRNA after 8 h of Arg starvation was detected exclusively in SAS cells (Fig. 2a; lower band). In FaDu cells, *sXBP1* accumulation occurred only after treatment with strong ER stress inducers such as tunicamycin or when ADT was combined with canavanine (Cav) as highlighted later. The data clearly demonstrate that the responder SAS but not the non-responder FaDu cells signal via the IRE1α-XBP1 pathway upon mono-ADT.

Activation of PERK or GCN2 kinases during ER stress suppresses global protein translation and activates ATF4-dependent translation through phosphorylation of eIF2α [31]. Mono-ADT resulted in the phosphorylation of eIF2α at Ser51 in both cell lines of interest with the same kinetics (Fig. 2b). Also, a constitutive time-dependent upregulation of the ATF4 gene and enhanced protein levels were observed (Fig. 2a/b). Prolonged mono-ADT further resulted in decreased phosphorylation and thus deactivation of the main mTOR target, ribosomal p70 S6 kinase, which participates in the cellular translation machinery (Fig. 2b).

The duration of eIF2α phosphorylation and translational inhibition depends on the activation of the protein phosphatase PP1, which dephosphorylates eIF2α, thereby restoring global mRNA translation [32, 33]. In this regard, a strong time-dependent induction of the corresponding *GADD34 *(*PPP1R15A*) gene expression was recorded only in SAS cells, whereas *GADD34* mRNA levels in FaDu cells initially increased and then gradually declined to basal levels at 24 h of Arg starvation (Fig. 2a). Figure 2a also documents that mono-ADT up-regulated the autophagy genes downstream of ATF4, e.g. *ATG12* and *SQSTM1* (*p62*), in SAS but not in FaDu cells; their products are the main components of autophagosome formation. Nonetheless, FaDu cells showed a severe treatment time-dependent formation of lipidated LC3-II protein, a well-known marker of autophagy, while SAS cells elicited high basal level of autophagy even without treatment (Fig. 2b).

Taken together, our data strongly suggest that mono-ADT triggers a massive and long-term ER stress induction, mainly via the IRE1α-sXBP1 and eIF2α-ATF4 signaling pathways, which is either exclusive or substantially more pronounced in the responder (SAS) than the non-responder (FaDu) cells.

### Mono-ADT-induced apoptosis is partially linked to ER stress

ER stress is inherently linked to apoptosis [17, 34]. Therefore, we monitored the activation of the main pro-survival and pro-apoptotic pathways in SAS and FaDu cells upon mono-ADT. First, we analyzed the level of phosphorylated (Ser473) Akt protein in Arg-starved cells and found a rapid increase in phosphorylated protein solely in SAS cells; the phosphorylation level of Akt protein in FaDu cells was unaffected (Fig. 2b). Similarly, the amount of phosphorylated Erk1/2 protein in SAS cells started to decrease 72 h after the onset of Arg starvation but did not systematically change in the FaDu model (Fig. 2b). These observations reveal that both Akt- and MAPK-mediated signaling pathways are involved in sustaining SAS cell survival upon mono-ADT.

One of the major factors contributing to ER stress-mediated cell death is CHOP [35, 36]. Expression of *CHOP* was gradually up-regulated upon mono-ADT in SAS cells. In contrast, the kinetics of *CHOP* gene expression in FaDu cells was similar to that of *GADD34* showing a decline within the first 24 h of Arg starvation (Fig. 2a). Unexpectedly, we noticed two peaks of induction for *ATF3* mRNA in SAS cells throughout the 120 h treatment period, whereas FaDu cells only showed the characteristic up-regulation at 2–4 h of Arg starvation followed by normalization of *ATF3* gene expression (Fig. 2a). This implies that the delayed but not early ATF3 response plays a role in stress-induced apoptosis during amino acid starvation [37, 38]. Accordingly, continuing Arg-withdrawal in the SAS cell model transcriptionally upregulated the Bcl-2 family members *BIM* and *BCL2* (Fig. 2a) and resulted in an accumulation of the caspase substrate poly(ADP-ribose) polymerase protein 1 cleaved form (cPARP) as well as Bim proteins 72 h after the onset of mono-ADT (Fig. 2b).

We next applied a chemical inhibitor of ER stress-induced apoptosis and selective siRNA-mediated knockdown approaches to further elucidate the link between ER stress and programmed cell death in Arg-deprived SAS cells. First, cells were exposed to the specific inhibitor of eIF2α dephosphorylation salubrinal (Sal, 0.02 mM) 1 h before and during mono-ADT to selectively block the main ER stress-induced apoptotic pathway [39]. WB analysis revealed that mono-ADT-dependent apoptosis in SAS cells reflected by cPARP protein is weaker (and/or delayed) and thus partially reduced in the presence of the ER stress modulator Sal (Fig. 2c). q-PCR analyses of several relevant genes to determine whether Sal pre-treatment diminishes global ER stress response in SAS cells are documented in Fig. 2e. 72 h of mono-ADT strongly enhanced *ATF3, ATF4, ATG12*, *GADD34* and *CHOP* gene expression (more than tenfold) in SAS cells; Sal pre-administration significantly inhibited the ADT-dependent upregulation of *ATF4, ATG12, ATF3* and *GADD34* gene expression. However, in parallel no reduction in the expression of *GRP78, CHOP, BAX*, *BID* and *BIM* genes was observed (Fig. 2e). This suggests that transcriptional upregulation of the Bcl-2 family members in SAS cells upon mono-ADT might be independent from ER stress response.

*IRE1, ATF4, GADD34,* and *CHOP* siRNA-mediated gene knockdowns were performed to identify potential ER stress-related mechanism involved in ADT-dependent apoptosis in the SAS model. As verified by q-PCR, transient transfection of siRNAs strongly down-regulated the expression of the respective target transcripts in the cells of interest (documented later in Fig. 5b). Knockdown of *IRE1* slightly enhanced the expression of cPARP protein upon mono-ADT (Fig. 2d). Interestingly, ATF4 protein expression and the phosphorylation level of eIF2α were also higher in *IRE1*-knockdown SAS cells. These data support the hypothesis that the IRE1α-XBP1 signaling arm is primarily a pro-survival pathway upon mono-ADT. Moreover, knockdown of the genes *ATF4* and *CHOP,* respectively, did not significantly alter mono-ADT-induced apoptosis in SAS cells (Fig. 2d). However, siRNA-mediated knockdown of *GADD34* significantly reduced the mono-ADT-dependent expression of cPARP and apoptosis induction which is in line with the Sal pre-treatment data.

Taken together, mono-ADT-related cell death in the responder SAS cells is not exclusively initiated via ER stress response and pro-apoptotic CHOP induction.

### Arg analog Cav strongly augments the efficacy of ADT = Comb-ADT

We previously reported that the Arg analog Cav is selectively cytotoxic at low doses for several cancer cell types under mono-ADT but not in an Arg-rich environment [9]. Importantly, Cav IC_50_ values of these cells were two orders of magnitude lower in Arg-deficient than Arg-rich media. Based on these IC_50_ data, we assayed the effect of 0.05–0.1 mM Cav on growth and regrowth capacity in SAS and FaDu monolayer cells. Both Cav concentrations showed very weak activity towards SAS and FaDu cells under Arg-rich conditions (Fig. 3a; Table S5A). By contrast, comb-ADT led to a significant reduction in the number of viable cells already after 24 h of treatment and resulted in a loss of regrowth capacity in both cell models. Accordingly, clonogenic potential in SAS and FaDu cells was unaffected by Cav under Arg-rich conditions but critically reduced after exposure to comb-ADT (Fig. S3B; Table S5B). Although SAS cells turned out to be more sensitive to comb-ADT than FaDu cells in all growth-related assays (Fig. 3a; S3B), Cav in principle strongly enhanced the ADT-induced growth-inhibitory and cytotoxic effects in both HNSCC models.

Clonogenic survival assays were applied to evaluate the impact of comb-ADT on the radioresponse. Colony formation capacity of SAS cells upon comb-ADT with 0.1 mM Cav dropped below detection level already without irradiation. Accordingly, the more sensitive SAS cells were treated with lower concentrations of Cav (0.01 mM and 0.05 mM), whereas FaDu cells were exposed to 0.05 mM and 0.1 mM Cav for 24 h prior to single dose X-ray (0–6 Gy). Cav alone in an Arg-rich environment did not affect clonogenic survival upon irradiation (Fig. S3A), but comb-ADT indeed strongly intensified the radioresponse and synergistically reduced clonogenic survival not only in the more radioresistant, responder model (SAS) but also in the intrinsic non-responder cell line FaDu (Fig. 3b).

### Comb-ADT results in catastrophic ER stress and apoptosis

q-PCR analyses of numerous genes of interest were performed to determine whether comb-ADT more efficiently triggers ER stress in HNSCC cells than mono-ADT. In an Arg-rich environment, 0.1 mM Cav did not alter the expression of various ER stress-related genes, neither in exponentially growing SAS nor in FaDu cells (Fig. 3c). As already demonstrated (Fig. 2a), 24 h of mono-ADT effectively upregulates *ATF3, ATF4, IRE1, GADD34* and *CHOP* gene expression in SAS and FaDu cells (Fig. 3c). Almost all of the ER response genes studied herein were more dramatically (factor > 10) enhanced by comb-ADT, e.g. *IRE1, ATF3, CHOP, GADD34*. In addition, comb-ADT triggered a strong splicing of *XBP1* mRNA not only in SAS but also in FaDu cells (Fig. S3C).

We next assessed comb-ADT-induced apoptosis in the two HNSCC cell line models and subsequently monitored the phosphorylation status of Erk1/2 MAPK and Akt protein kinases. A progressive accumulation of cPARP protein and apoptosis induction, respectively, was clearly observed in SAS (more pronounced) and FaDu cells after 24 h of comb-ADT (Fig. 3d). The levels of p-Akt also increased during comb-ADT in both cell types, while the phosphorylation of Erk1/2 protein was differentially altered by the treatment (Fig. 3d).

Gene expression analyses revealed that most of the ER stress response genes enhanced during comb-ADT (e.g.* ATF3, GADD34, CHOP, IRE1, GRP78*) were less affected in SAS and FaDu cells conditioned with Sal (0.02 mM) (Fig. 3c). Moreover, Sal pre-exposure reduced the comb-ADT-dependent transcriptional upregulation of the pro-apoptotic genes *BID* and *BIM*, and it also considerably attenuated the comb-ADT-induced expression of cPARP protein in both HNSCC cell models (Fig. 3d). Conventional MTT cell viability assays further confirmed that Sal partially protected the cells from comb-ADT-related cytotoxicity (Fig. 3e); this protective effect was higher in the sensitive SAS cells. Nonetheless, comb-ADT was in principle found to cause catastrophic ER stress linked to apoptosis induction in both HNSCC cell lines.

### eIF2α-ATF4(GADD34)-CHOP pathway is involved in apoptosis upon Comb-ADT

**Fig. 4:**
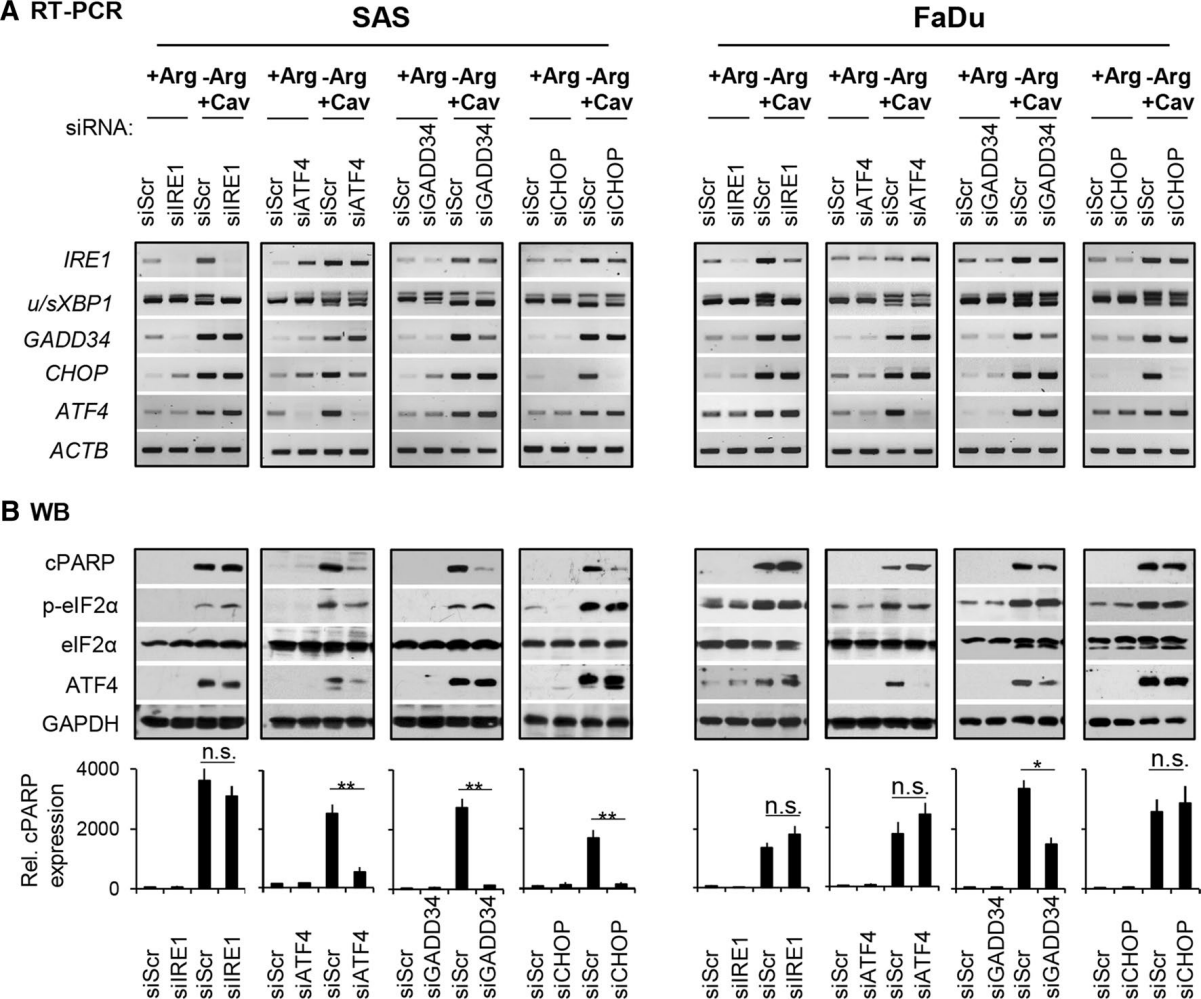
eIF2α-ATF4(GADD34)-CHOP pathway is involved in apoptosis upon comb-ADT. Representative results of *N* = 3 independent experiments in SAS and FaDu 2-D cultures are documented; cells were transfected with the respective siRNAs according to Materials and Methods, comb-ADT was achieved by exposure to − Arg medium supplemented with 0.1 mM Cav. **a** RT-PCR data sets showing the expression of several ER stress response genes in siRNA-mediated knockdown cells exposed for 24 h to comb-ADT or control conditions. **b** Western blots illustrating proteins of interest in siRNA-mediated knockdown cells exposed to comb-ADT or control conditions for 24 h (top) and densitometric analysis of the relative cPARP expression normalized to GAPDH (bottom); *p ≤ 0.05; **p ≤ 0.01; *n.s.* not statistically significant

To unravel the comb-ADT-induced ER stress–apoptosis axis, we studied the role of several upregulated genes upon treatment in more detail via diverse knockdown experiments. The downregulation of the different ER stress genes of interests in all siRNA-mediated knockdown cells compared to the respective scrambled siRNA control cells are documented in Fig. 4a. Notably, knockdown of *ATF4* induced an upregulation of *IRE1* and *sXBP1* as well as *GADD34* genes in both cell types, suggesting that the cells can intrinsically compensate stress responses via an alternative IRE1α-sXBP1 pathway. We also found cPARP protein upon comb-ADT to be unaffected or only slightly enhanced upon knockdown of *IRE1* in both cell lines (Fig. 4b). Notably, the levels of ATF4 and p-eIF2α proteins, as well as *GADD34* and *CHOP* genes were also unaltered in the siIRE1 knockdown cells (Fig. 4a/b). It is, therefore, likely that activation of the IRE1α-XBP1 pathway during comb-ADT constitutes a pro-survival response.

Interestingly, siRNA-mediated downregulation of the genes *ATF4* and *CHOP* systematically lowered comb-ADT-induced apoptosis as reflected by reduced cPARP levels only in SAS cells but not in FaDu cells (Fig. 4b). However, in line with the Sal protection data described in the previous chapter, knockdown of *GADD34* basically reduced comb-ADT-dependent apoptosis in both cell models, notwithstanding that the effect was more pronounced in SAS cells. The integrated data thus clearly indicate that the eIF2α-ATF4(GADD34)-CHOP pathway plays a role in HNSCC cell death caused by comb-ADT.

### Mono-ADT-induced radiosensitization is linked to ER stress response but mechanistically differs from Comb-ADT

Clonogenic survival after single dose irradiation (0–8 Gy) was monitored under −Arg conditions (i) when (pre)-exposed to either Sal (0.02 mM) or to dimethyl sulfoxide (DMSO, 2%) as ER stress modulators, or (ii) upon defined siRNA-mediated gene knockdown. The aim of this experimental setup was to demonstrate a causal relation between ER stress response and the selective radiosensitization triggered in the SAS cell model by mono-ADT.

**Fig. 5:**
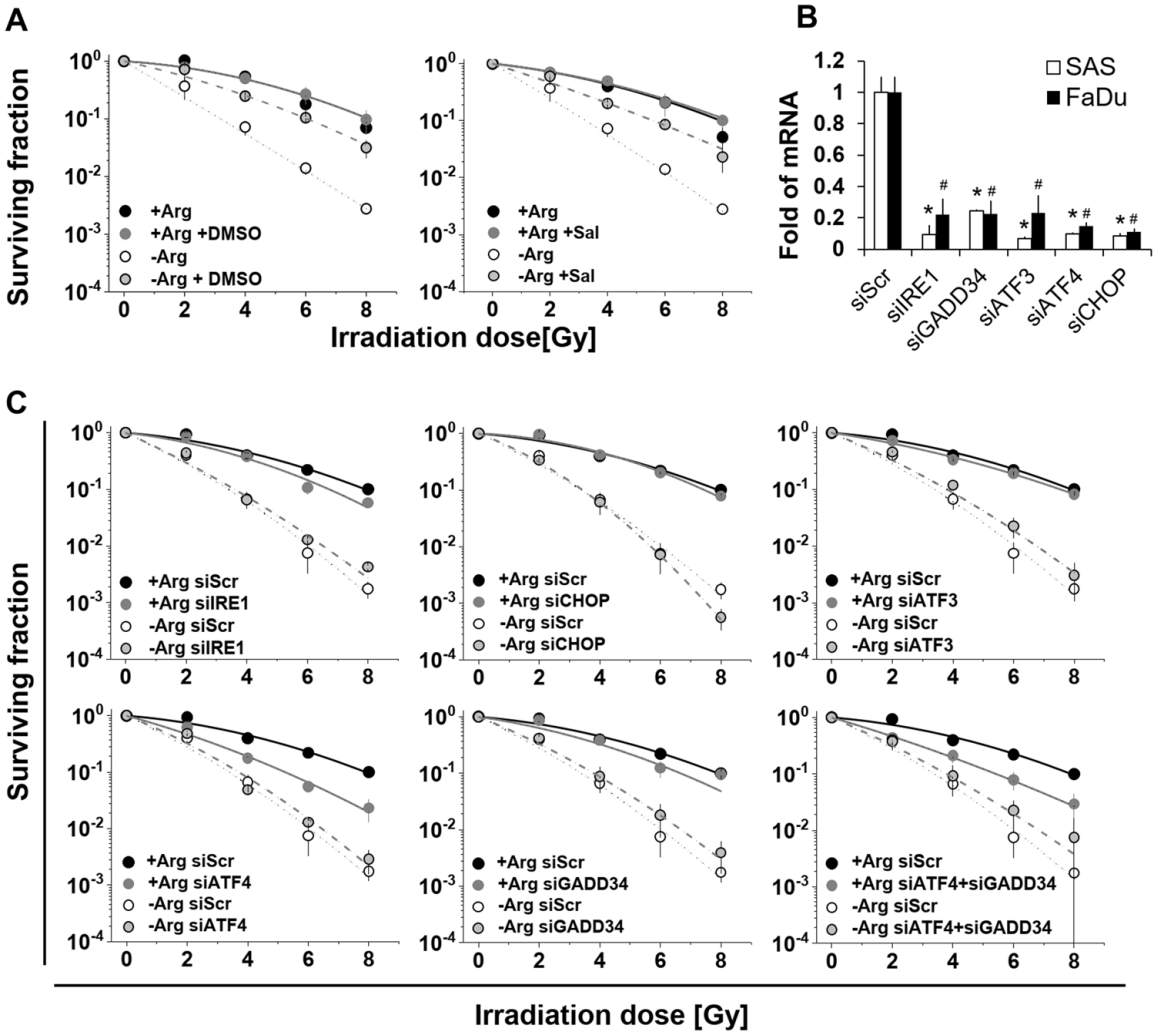
Role of ER stress in mono-ADT-induced radiosensitization mechanistically differs from comb-ADT. All graphs in **a**/**b** show mean surviving fractions (± SD) from three independent experiments in 2-D culture each with ≥ 3 biological replicates (*N* = 3, *n* ≥ 3); data were fitted according to Fig. 1. **a** Clonogenic survival of SAS cells irradiated with single doses of 0–8 Gy under either standard conditions or upon 24 h pre-exposure to Arg-free medium with and without Sal (0.02 mM) or dimethyl sulfoxide (2% DMSO). **b** Documentation of qPCR analysis reveals sufficient knockdown of genes of interest in SAS and FaDu cells 3 days after transfection with the respective siRNAs or scrambled control (siScr). *p ≤ 0.01 significant in SAS cells; ^#^*p* ≤ 0.01 significant in FaDu cells. **c** Clonogenic survival of siRNA-mediated knockdown and siScr control SAS cells irradiated with single doses of 0–8 Gy under either standard conditions or upon 24 h pre-exposure to Arg-free (− Arg) medium.

DMSO is an efficient disaggregating agent known to prevent protein aggregation, thereby reducing ER stress signalling [11, 40]. Notably, DMSO at the designated concentration possessed general radioprotective activity even in a + Arg environment (Fig. 5a, Table S6A). More importantly, however, both DMSO and Sal significantly diminished the radiosensitizing effect of mono-ADT (Fig. 5a), indicating a causal link between radiosensitization and ER stress response. We next recorded the radioresponse of SAS cells upon knockdown of the genes *IRE1, ATF4, GADD34, ATF3* and *CHOP*. The efficiency of gene knockdown in the respective experiments is documented in Fig. 5b. Except for the knockdown of *ATF4*, transient transfection of siRNAs did not significantly alter the surviving fractions of SAS cells after irradiation in Arg-rich medium (Fig. 5c; Table S6B). *ATF4* knockdown alone or in combination with *GADD34* knockdown resulted in a slight, yet significant, sensitization of SAS cells to radiation under + Arg conditions. This finding implies that the ATF4 pathway is an inherent component in the radioresponse of SAS cells. At the same time, however, knockdown of none of these ER stress genes of interest systematically or significantly altered the radiosensitizing effect of mono-ADT (Fig. 5c; Table S6B).

To conclude, the causal link between mono-ADT-induced radiosensitization and ER stress remains an open case but clearly differs mechanistically from the ER stress-related cell response to comb-ADT.

### Mono- and comb-ADT-induced growth arrest and radiosensitization are revealed in 3-D models

**Fig. 6:**
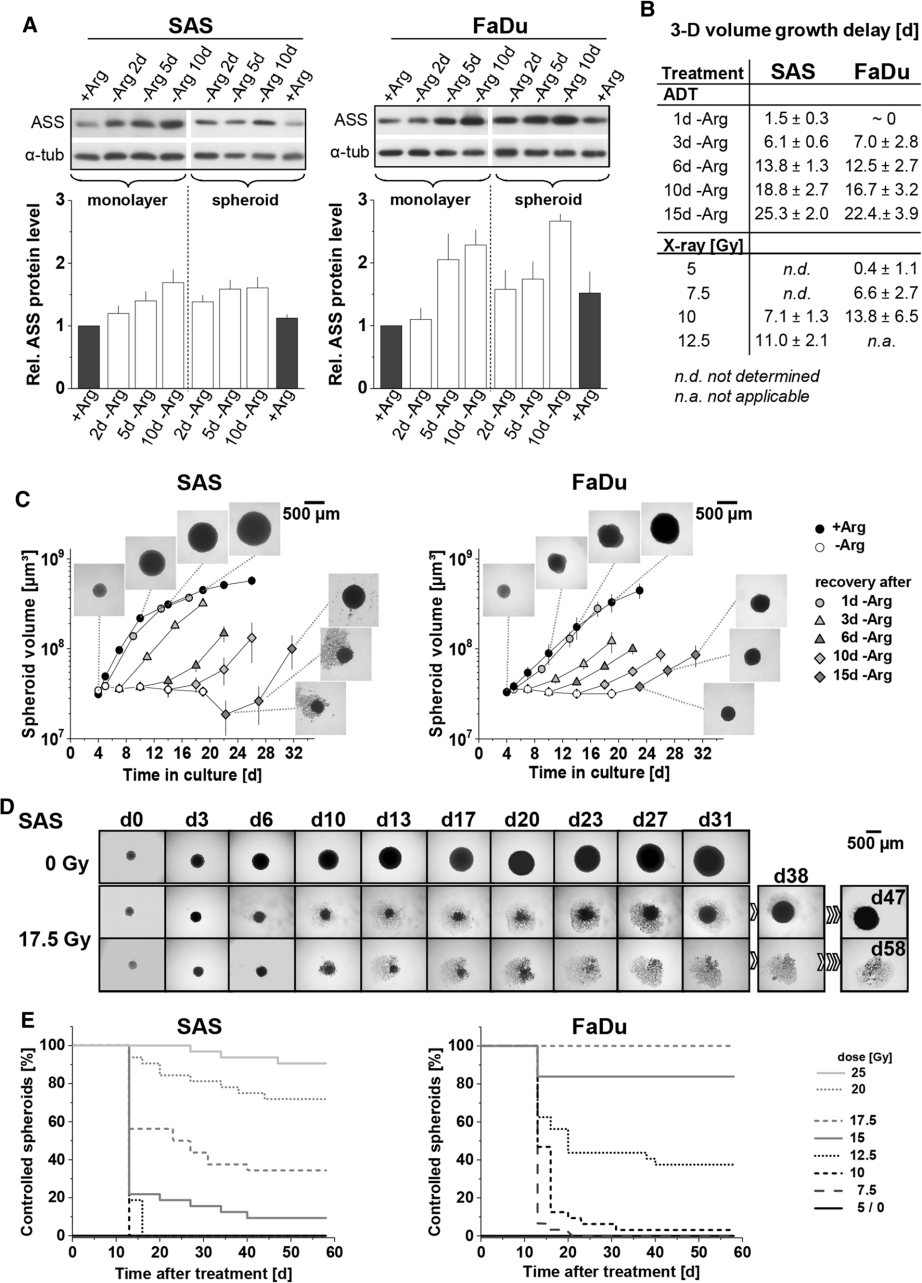
HNSCC 3-D cultures are less susceptible to mono-ADT despite an acute growth arrest; 3-D assay well reflects the different radioresponse of SAS and FaDu cells and xenografts (cf. Fig. S2B). **a** Representative western blots of ASS protein from 2-D and 3-D cultures (SAS, FaDu) grown under − Arg conditions for 2–10 days. Densitometrical analysis of the relative ASS expression normalized to α-tubulin are presented as means (± SD) from *N* = 3 independent experiments. **b** Volume growth delay (in days) in SAS and FaDu spheroids after different times of Arg-starvation or single dose irradiation with up to 12.5 and 10 Gy, respectively. **c** Volume growth kinetics of SAS and FaDu spheroids with and without mono-ADT (1–15 days). After termination of treatment, spheroids were re-transferred into standard culture medium to also assess 3-D regrowth capacity and kinetics. **d** Image series of representative SAS spheroids without (0 Gy) and after treatment with 17.5 Gy single dose X-ray to illustrate readout and analytical endpoint of the spheroid control probability (SCP) assay. A regrown and a controlled spheroid in the treatment arm are shown as examples; the routine monitoring time was 60 days post-treatment. **e** Proportion of controlled SAS and FaDu spheroids after 5–25 Gy single dose irradiation based on ≥ 30 spheroids monitored per treatment arm and documented as function of time post-treatment according to **d**

It was described earlier that a three-dimensional environment renders colorectal cancer cells less susceptible to single amino acid starvation [41]. We, therefore, intended to prove the efficacy of ADT in HNSCC cells in a 3-D cellular context using SAS and FaDu spheroid models. First, ASS status was assessed in whole cell protein lysates from 2-D and 3-D cultures kept in regular + Arg conditions and upon 2–10 days of Arg deprivation. In spite of different basic ASS protein level in FaDu and SAS cells, densitometric analysis of WB revealed systematically increasing ASS as function of time throughout mono-ADT for both cell lines in 2-D as well as in 3-D culture (Fig. 6a).

Both FaDu and SAS spheroids stopped growing after transfer into -Arg (Cit supplemented) media but basically remained intact and started to regrow upon re-transfer into a + Arg environment even after 15 days of starvation in contrast to the more sensitive monolayer cultures (Fig. 6C). The sensitivity loss in the 3-D environment correlates with a less prominent ER stress induction under these conditions (Fig. S4B/C). Monitoring of volume growth kinetics and evaluation of the time to reach the 5-times spheroid volume before treatment (5 × *V*_0_) relative to + Arg control spheroids allowed to determine a spheroid volume growth delay for each treatment arm and period, respectively. Data reveal that spheroid volume growth was slowed (FaDu) or regrowth was increasingly delayed (SAS with lag phase) with incremental periods of mono-ADT (Fig. 6b/c).

Radioresponse in spheroids was evaluated either by analyzing volume growth delay in case of 100% spheroid regrowth, i.e. after lower doses of X-rays (Figs. 6b and S4A), or more importantly by monitoring all spheroids over a period of up to 60 days post-treatment, thereby determining the spheroid control probability (SCP) as the proportion of controlled (non-regrown) spheroids as function of time (Fig. 6d/e). The SCP assay used herein allows to determine the analytical endpoint SCD_50_ (spheroid control dose 50) which is the irradiation dose leading to 50% of controlled spheroids in analogy to the tumor control probability and TCD_50_ in vivo assay as highlighted earlier [15]. First signs of controlled spheroids were seen at doses of 15 Gy and 10 Gy for SAS and FaDu spheroids, respectively. The SCD_50_ values were 18.8 Gy for SAS and 13.0 Gy for FaDu spheroids which is in line with the higher intrinsic radioresistance of SAS cells documented earlier in the respective tumor xenograft models [20].

**Fig. 7:**
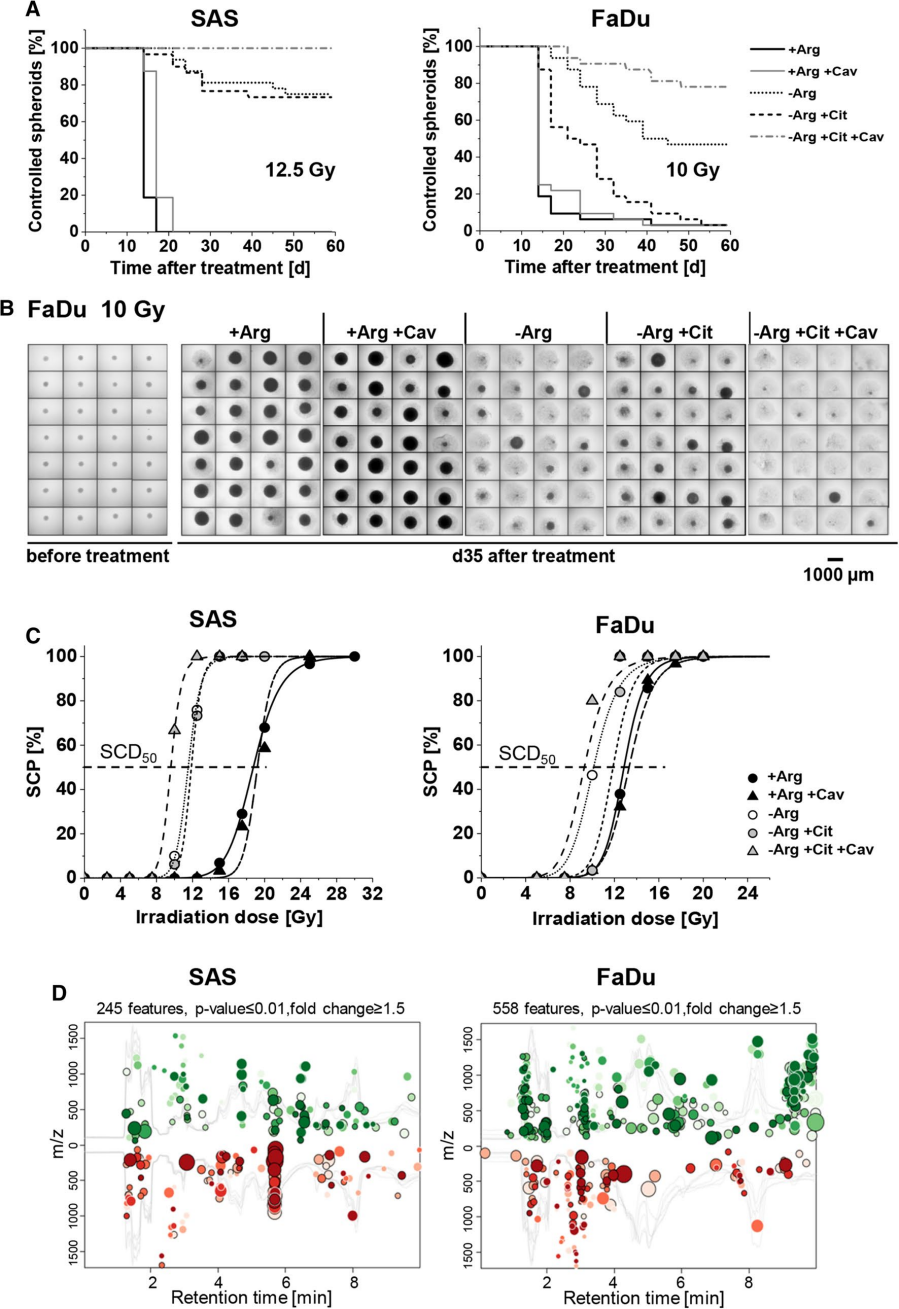
Mono-ADT mainly radiosensitizes the metabolically less flexible, radioresistant SAS 3-D cultures while comb-ADT evokes a supra-additive radiosensitizing effect in both SAS and FaDu models. In the SCP assays, a minimum of 30 individual spheroids were monitored in each treatment arm over a 60-day post-treatment period (cf. Fig. 5e). **a** Proportions of controlled spheroids as function of time post-treatment with mono- or comb-ADT (1 day) plus single dose irradiation with 12.5 Gy for more radioresistant SAS spheroids and 10 Gy for FaDu spheroids. **b** Representative images of FaDu spheroids before and at 35 days post-treatment with comb-ADT and 10 Gy X-ray to illustrate the efficacy of combinatorial treatment. **c** Spheroid control dose response curves after 0–30 Gy single dose irradiation for spheroids with or without 24 h pre-exposure to − Arg medium in the absence or presence of 0.04 mM Cit and 0.1 mM Cav. **d** Interactive cloud plots (generated with the XCMS Online platform) visualizing diverse significantly regulated global metabolic features in SAS and FaDu spheroids upon 24 h mono-ADT. Color intensities reflect *p* values and the dot sizes represent the fold change. Plots are produced from *N* = 2 independent experiments with up to six technical replicates

Figure 7a–c clearly document that mono-ADT leads to radiosensitization in HNSCC spheroids and is augmented by comb-ADT. SCD_50_ values after mono-ADT were significantly lowered relative to + Arg control spheroids (Table S7A/B). In contrast to 2-D culture, slight radiosensitization was also seen in the putative non-responder FaDu model. However, this effect was much smaller than in SAS spheroids and severely impeded—yet not entirely abolished—in the presence of 0.04 mM Cit resulting in mean dose reduction factor (DRF) values of 1.6 for SAS and only 1.1 for FaDu spheroids. Application of 0.1 mM Cav alone appeared to be ineffective showing no radiosensitizing potential; comb-ADT, however, had a clear synergistic impact on radioresponse resulting in SCD_50_ values as low as 9.6 Gy for SAS and 9.3 Gy for FaDu spheroids. Thus, radiosensitivity was significantly enhanced in SAS and FaDu spheroids upon comb-ADT reflected by mean DRFs of 1.9 and 1.4, respectively. SCD_50_ and DRF values with 95% confidence intervals and statistical evaluation for all treatment arms are listed in Table S7A/B.

### Mono-ADT results in analogous and differential metabolic modifications in HNSCC spheroids

We performed metabolomics profiling to gain first insight into global and differential metabolic alterations induced by mono-ADT in the two HNSCC spheroid models. A targeted GCxGC-MS method [25] allowed us to identify a total of 72 amino-acid-related metabolites in treated and untreated SAS and FaDu spheroids. In brief, we found 11 amino acids including alanine, serine, threonine, leucine and lysine as well as glycerol to be significantly (or tendentially) enhanced upon mono-ADT in both models (Fig. S5B). This is indicative for a general protein and membrane breakdown resulting from Arg deficiency. Alterations in intracellular free amino acids were in most cases more pronounced in the non-responder FaDu model. Three metabolic features appeared to be differentially affected in the two HNSCC spheroid types, namely urea, phosphoric acid and glutaric acid (Fig. S5C). Urea was reproducibly enhanced only in the treated versus untreated SAS cells while changes in phosphoric acid and glutaric acid levels rather manifested in the FaDu model.

LC–MS-TOF-based untargeted metabolomic profiling was performed to assess global metabolome changes. The respective data documented in Fig. 7d indicate a higher metabolic plasticity in the non-responder FaDu cells with almost 560 metabolic characteristics being critically altered upon mono-ADT, whereas the responder SAS cells showed modifications at a similar significance level only in < 250 metabolic features. Having said that, the cloud blot also illustrates that different metabolites appear to be affected in the two cell models. The number of metabolites lowered after mono-ADT is proportionally much higher in the responder SAS than in the FaDu cells; the striking depletion of numerous metabolites detected in the retention time period of 5.5–5.8 min which is clearly missing in poor/non-responder FaDu cells shall be highlighted. The particular treatment-induced increase in metabolome signals at early (1.2–1.5 min) and very late (> 9 min) retention times in the FaDu model are another, antidromic example (Fig. 7d).

## Discussion

To this day, anti-EGFR treatment with cetuximab has been the only targeted therapy shown to substantially improve HNSCC patient outcome, although its superiority over Cisplatin when combined with irradiation as first-line therapy for locally advanced, non-metastatic HNSCC is increasingly questioned based on recent clinical data [42]. Nonetheless, other molecular targeting approaches have had little success and only cetuximab still remains to be approved by the FDA for multimodal therapy in patients with refractory and/or metastatic HNSCC and as radiosensitizer in combination with chemoradiation for patients with locally advanced disease [18]. Studies on the potential of metabolic targeting strategies considered for HNSCC patients mainly focus on the identification and development of hypoxia modifiers such as nimorazole, which is in clinical trial as radiosensitizers [43, 44]. Other metabolic targeting approaches such as enzymotherapeutic ADT have not yet been considered in multimodal HNSCC treatment, mainly because of general lack of preclinical data and mechanistic insight for arginine non-auxotrophic cancers.

Although mono-ADT was shown earlier to exhibit some anticancer efficacy in vitro against human laryngeal squamous cell carcinoma cells [45], HNSCC do not belong to the proposed group of Arg-auxotrophic (responding) cancers because the key enzymes for converting Cit to Arg, in particular ASS, are in principle expressed in these tumor cells. Indeed, the *ASS1* mRNA levels appears to be significantly upregulated in ~ 9% of primary HNSCC and a strong negative correlation between high tumor *ASS1* levels and clinical outcome (relapse-free and overall survival) is observed in the HNSCC patient cohort of The Cancer Genome Atlas (TCGA) dataset. According to this data (Fig. S2) and previous findings in oral squamous cell carcinomas [46], the high level of *ASS1* seems to be a reliable independent prognostic marker of poor disease-free survival in HNSCC patients. Moreover, our data also revealed a potential intermediate correlation between ASS1 expression level and radioresistance in HNSCC cell lines in vitro. Nonetheless, this observation was not indicative for mono-ADT-induced radiosensitization suggesting that some patients with HNSCC or other aggressive non-auxotrophic cancers might indeed benefit from ADT if applied in combinatorial treatment regimes and upon patient stratification.

As expected, the lack of Arg led to a strict growth arrest in all tested HNSCC cells in 2-D culture. However, the exogenous Arg precursor Cit, which is basically present in the blood stream and tissue interstitium, partially promotes growth under-Arg conditions and rescues regrowth capacity in the majority of HPV^−^ HNSCC cell lines upon Arg re-supplementation. Indeed, cell-line specific sensitivities to mono-ADT mainly manifest in differences in re-growth capacity after completion of the treatment and in the response to Cit under Arg-deprived conditions. All HNSCC cell lines express the Arg biosynthetic enzyme ASS and are also positive for ASL. However, neither the expression levels nor the induction of ASS in the absence of Arg correlate with the cells’ sensitivity to mono-ADT indicating that the expression of ASS is invalid surrogate marker for treatment response and thus also for patient stratification. Subsequently, we in detail examined whether—and how—prolonged mono-ADT induces metabolic stress, in particular ER stress, to gain insight into other molecular mechanisms that might determine the cells’ response to ADT.

ER stress pathways in mammalian cells are induced by three specific signaling cascades mediated by primary ER-localized protein stress sensors: IRE1α (inositol-requiring enzyme 1 alpha), PERK (double-strand RNA-activated protein kinase-like ER kinase), and ATF6 [17, 47, 48]. Upon activation and to restore protein homeostasis, all primary sensors induce signal transduction processes that increase the levels of ER chaperones and inhibit protein entry into the ER by arresting mRNA translation. We found here that mono-ADT triggers a massive and durable ER stress in the more sensitive SAS cells with strong expression of the majority of ER stress response markers, such as *IRE1, sXBP1, ATF4, GADD34, CHOP*, etc. By contrast, only activation of short-term and mild ER stress upon mono-ADT was observed in the conditionally resistant FaDu cells. The most striking differences between responder and non-responder cell models was seen in the activation of the IRE1α-sXBP1 and eIF2α-ATF4-GADD34-(CHOP) pathways as well as their targets. The obtained data suggest that long-term Arg deficiency for yet unknown reasons does not induce massive and acute ER stress in resistant FaDu cells.

ER stress may induce both pro-survival adaptive and apoptotic responses depending on the severity and durations of the ER stress [17, 48]. ATF4 and ATF6 regulate *CHOP* gene expression to trigger apoptosis [35]. These factors as well as apoptosis were induced upon long-term mono-ADT selectively in the responder SAS cell line. However, knockdown of the pro-apoptotic CHOP molecule did not rescue SAS cells from apoptosis, suggesting that the CHOP-mediated pathway most probably is not involved in the mono-ADT-induced ER-stress-related cell death of the responder cells. The same holds true for the IRE1α-XBP1 signaling arm. However, our data clearly reveal that inhibition of GADD34 by Sal or specific siRNA knockdown partially protects SAS cells from mono-ADT-dependent apoptosis. We therefore assume that the profoundly up-regulated GADD34 in SAS cells dephosphorylates eIF2α, abolishes translational inhibition and contributes to further accumulation of unfolded proteins in the ER, thereby also allowing translation of mRNAs encoding pro-apoptotic proteins [32, 33]. In conclusion, ER stress is involved but is not a single player in the multifactorial process contributing to cancer cell death upon mono-ADT.

Cav, a natural Arg proteomimetic analog of plant origin has been shown at low concentration to selectively inhibit proliferation, evokes cell death and eventually sensitize cancer cells to irradiation upon ADT [9, 10, 12, 13]. Cav is taken up by cells via the same amino acid transporters as Arg [49]. Its anti-cancer efficacy has been linked to its capability to incorporate into cellular proteins, rendering them misfolded or non-functional thereby inducing ER stress [9, 11, 14]. We demonstrated earlier that the IC_50_ values of Cav in Arg-free conditions were one order of magnitude lower in various cancer cell lines than in three (pseudo)normal cell types; the latter were also capable of restoring their proliferation upon comb-ADT in contrast to the more sensitive cancer cells [9]. Experiments with a sophisticated 3-D organoid culture of primary human colon epithelium further showed that the combination of an Arg-deprived environment with low dose Cav treatment did not affect normal colonosphere integrity or result in PARP fragmentation as seen in colon cancer cell line organoids [13]. To underline these previous findings, we also applied 3-D cultures originated from various other normal cell types, i.e. normal skin fibroblasts, HUVEC and the retinal pigment epithelial cell line ARPE-19. The latter produced discoid aggregates only (Fig. S6A), while fibroblast and HUVEC formed more round-shaped non-growing spheroid cultures (Fig. 6b). We found neither Arg-deprivation nor up to 0.1 mM Cav in the presence or absence of Arg to systematically reduce the viability of any of these cell models in the 3-D environment (Fig. S6). These data further support the hypothesis of a putative therapeutic window for mono- and comb-ADT approaches, although in vivo application will be critical for further evaluation and approval.

In the HNSCC cells, Cav under ADT conditions clearly induced ER stress and apoptosis. Mechanistically, Cav in the absence of Arg evoked a strong activation of the IRE1α-sXBP1 pathway and of downstream mediators in the eiF2a-ATF4-GADD34(CHOP) signaling axis. Moreover, the inhibition of GADD34 diminished comb-ADT-induced apoptosis in HNSCC cells, indicating that the two events are linked. Our siRNA-mediated knockdown analyses proved that the IRE1α-sXBP1 pathway as for mono-ADT does not play a significant role in cell death upon comb-ADT. In contrast, GADD34, CHOP and ATF4 molecules turned out to be required for comb-ADT-mediated apoptosis in the responder SAS cells. Thus, Cav at low doses clearly exacerbates the ER stress and pro-apoptotic effects induced by ADT in HNSCC cells.

As the majority of HNSCC patients receive radiotherapy during the course of their disease, we also combined ADT with X-ray irradiation to identify a putative radiosensitizing potential of this treatment option. We are the first to report that more than 50% of ASS1-positive HPV-negative HNSCC cell lines are indeed radiosensitized by mono-ADT in the presence of physiological Cit concentration. It is known that exposure to ionizing radiation induces ER stress in cancer cells [48, 50, 51]. One of the pathways of ER stress response, the PERK-eIF2α pathway, was for example shown to modulate radiosensitivity in breast cancer cells [52, 53]. Moreover, chemical ER stress inducers, such as tunicamycin or thapsigargin, enhanced irradiation-induced caspase 3 activation and DNA fragmentation in intestinal epithelial cells [51]. In the present study, we found the intrinsic radioresistance of SAS cells to be reduced upon ATF4 knockdown indicating an ATF4-dependent pro-survival mechanism in these HNSCC cells. On the other hand, mono-ADT-induced radiosensitization in SAS cells was significantly diminished by the ER-stress modifiers pointing at a causal link between ER stress and radiosensitization. However, the observation that selective siRNA-mediated knockdowns of *IRE1, ATF3, CHOP* and *GADD34* (as well as ATF4) did not modulate the radiosensitizing effect implies a different and even more complex underlying mechanistic scenario as compared to the apoptosis induction by comb-ADT which is clearly mediated in the same cells via the eiF2a-ATF4-GADD34(CHOP) ER stress response signaling pathway.

ATF4 seems to be a pro-survival factor in radioresistant SAS cells, but is also involved in pro-apoptotic signaling when these cells are exposed to ADT in combination with Cav. We, therefore, hypothesized that Cav co-application might switch the ATF4 function resulting in an ADT-synergistic radiosensitizing effect. The latter was indeed proven even in our sophisticated 3-D radioresponse assay when combining ADT with Cav and irradiation. Low doses of Cav clearly enhanced the radiosensitizing potential of ADT in the SAS model and also induced radiosensitization in the initial non-responder FaDu cells both in a 2-D and 3-D environment.

Global metabolic stress and metabolic features of treated HNSCC cells were also recognized in targeted and untargeted metabolomics data. Mono-ADT in general was accompanied by elevated intracellular free amino acids and glycerol as indicators of protein and membrane breakdown. Metabolic modifications seen in both responder and non-responder cells as well as those exclusively related to rhARG-dependent degradation of Arg such as urea accumulation are unlikely to play a role in cell specific radiosensitization as the latter is also seen under dietary-Arg conditions. However, overall the metabolic landscape appeared to be less plastic and more negatively affected by ADT in the responder SAS model which is ultimately more sensitized to combinatorial treatments than the FaDu model. Whether ADT-induced metabolic limitations enhance the susceptibility of SAS cells to irradiation and Cav co-application or the higher metabolic plasticity in the conditionally resistant FaDu cells reflects particular prosurvival adaptations remains to be elucidated. Yet, the different metabolic dynamics of the two HNSCC models upon treatment suggest complex cellular stress responses beyond the ER.

Notably, other published data suggest that metabolic stress induced by ADT may not only result in autophagy and/or cell death pathways triggered by ER stress response. ADI-PEG20 treatment in prostate cancer cells, for example, critically enhanced ROS generation and impaired mitochondrial function as well as DNA integrity; here, the ROS scavenger N-acetyl cysteine (NAC) could abolish the ADI-PEG -induced effects [54]. A similar response to ADI-PEG20 with respect to ROS generation and mitochondrial dysfunction was reported in ASS1-deficient breast cancer cells [55]. This was revealed in a more recent study showing that Arg is essential for proper mitochondrial respiration and critical for cellular acetyl-CoA level to maintain the H3K9 acetylation in the ASS-deficient breast cancer cells [56]. In these cells, ADT reduced glycolytic flux, down-regulated mitochondrial metabolites, inhibited OxPhos via gene regulation, increased ROS production and initiated cell death, while cells lacking mitochondria were resistant to the effects of Arg depletion [56]. ROS are established key modulators in radioresponse (e.g. [57]) and OxPhos inhibition is increasingly recognized in recent years as a strategy to sensitize particular malignant cells to radiotherapy (e.g. [58]), indicating that mitochondrial dysfunction can critically contribute to the radiosensitizing potential of ADT. The relevance of this proposed causal link in ASS1 non-deficient cancers is unclear and shall thus be assessed next in our well-characterized 3-D HNSCC models.

## Conclusions

**Fig. 8:**
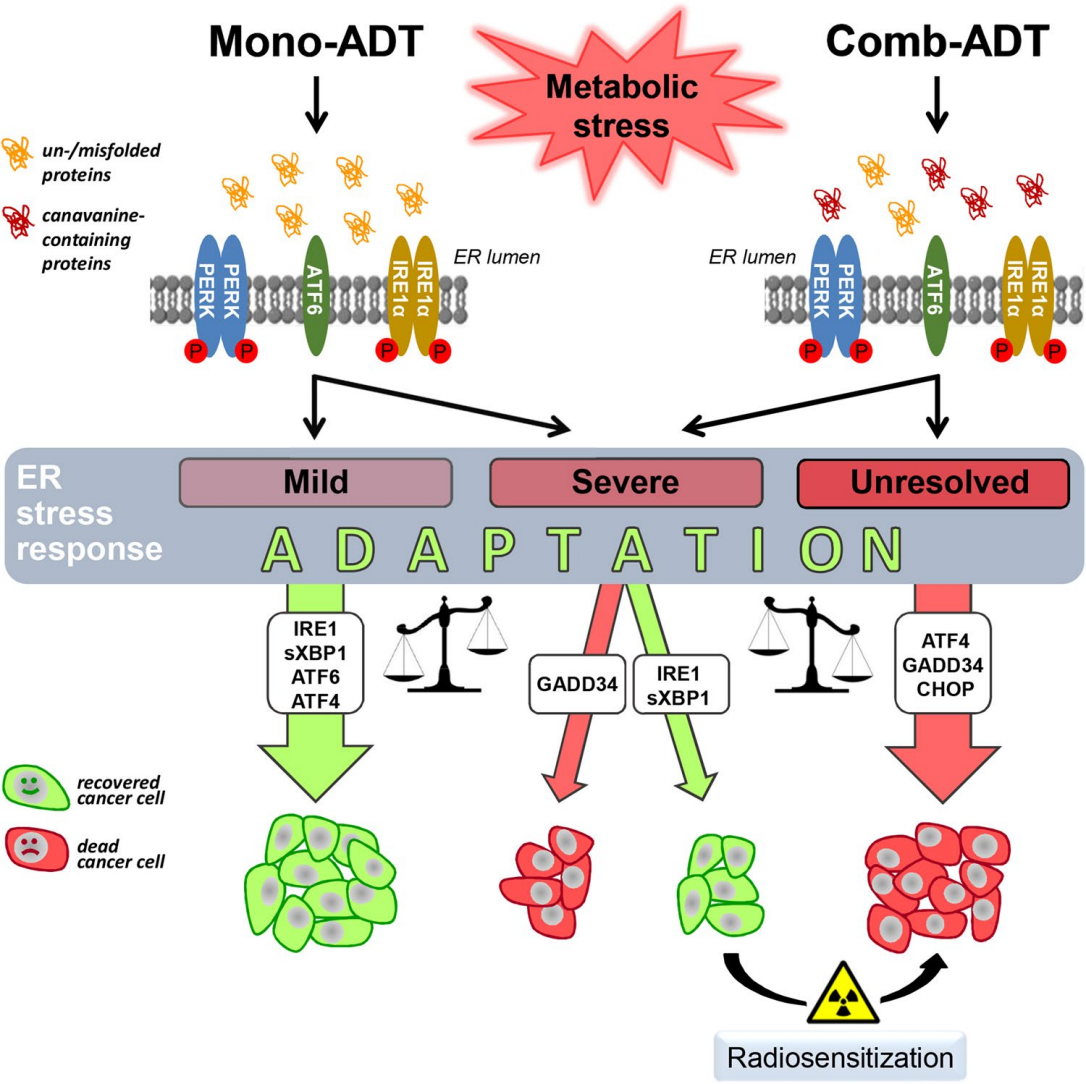
Schematic illustration of the proposed role of ER stress pathways in mono-ADT and comb-ADT as well as in ADT-induced radiosensitization

In summary, the extensive data set of our study implies that ER stress pathways play a dualistic role in the cells’ response to ADT depending on their individual intrinsic sensitivity to the triggered ER stress, its severity and duration (Fig. 8). For example, mono-ADT-induced a strong ER stress response only in responsive SAS cells but not in non-responder FaDu cells. However, comb-ADT with Cav caused a catastrophic ER stress response and ER stress-mediated apoptosis in both models and elicited a supra-additive effect with respect to radiosensitization. Notably, many natural and synthetic chemical drugs targeting ER stress response pathways are currently under investigation in pre-clinical and clinical trials [48, 59, 60]. Their application to enhance the anticancer efficacy of ADT and improve the curative outcome of radiotherapy is envisioned and should explicitly be explored in vitro and in vivo in parallel to extended mechanistic studies that need to consider the functional analysis of various intracellular organelles including not only ER but also mitochondria. We propose that the combination of ADT with low doses of Cav and/or selective ER stress modulators provides a promising new strategy for radiosensitization of Arg-auxotrophic, but also intrinsically non-auxotrophic cancers such as HNSCC.

## Electronic supplementary material

Below is the link to the electronic supplementary material.

Supplementary file1 (PDF 751 KB)

Supplementary file2 (PDF 1345 KB)

## Abbreviations

ADT: Arginine deprivation therapy
Akt: Protein kinase B
Arg: Arginine
ASS: Argininosuccinate synthetase
ATF3: Activating transcription factor 3
ATF4: Activating transcription factor 4
ATF6: Activating transcription factor 6
Atg12: Autophagy related 12
Bax: Bcl-2-associated X protein
Bcl-2: B-cell lymphoma 2
Bim: Bcl-2-like protein 11
Cav: Canavanine
CHOP: C/EBP homologous protein
Cit: Citrulline
comb-ADT: Arginine deprivation therapy combined with canavanine
CPD: Cumulative population doublings
cPARP: Cleved poly(ADP-ribose) polymerase
DMEM: Dulbecco’s modified eagle medium
DMSO: Dimethyl sulfoxide
DRF: Dose reduction factor
EDTA: Ethylenediaminetetraacetic acid
eIF2α: Eukaryotic translation initiation factor 2α
ER stress: Endoplasmic reticulum stress
Erk1/2: Extracellular signal-regulated kinase 1/2
ESI: Electrospray ionization
FCS: Fetal calf serum
GADD34 (PPP1R15A): Growth arrest and DNA damage-inducible protein
GAPDH: Glyceraldehyde-3-phosphate dehydrogenase
GCxGC-Qms: High-speed scan single quadrupole mass spectrometry
Grp78 (HSPA5): 78-KDa glucose-regulated protein
HEPES: 4-(2-Hydroxyethyl)-1-piperazineethanesulfonic acid
HNSCC: Head and neck squamous cell carcinoma
HPV: Papillomavirus
IRE1α: Inositol-requiring enzyme 1α
LC3-II: Microtubule-associated protein 1A/1B-light chain 3
LC–MS: Liquid chromatography mass spectrometry
MAPK: Mitogen-activated protein kinase
mono-ADT: Arginine deprivation mono-therapy
mTOR: Mammalian target of rapamycin
PBS: Phosphate-buffered saline
PERK: Protein kinase R (PKR)-like endoplasmic reticulum kinase
PP1: Protein phosphatase 1
q-PCR: Real-time quantitative polymerase chain reaction
rhARG: Recombinant human arginase
RT-PCR: Reverse transcription polymerase chain reaction
Sal: Salubrinal
SCD_50_: Spheroid control dose 50
SCP: Spheroid control probability
SFD: Surviving fraction dose
siRNA: Small interfering RNA
SQSTM1 (p62): Sequestosome-1
SUMO: Small utilities of microarray data analysis
TCD_50_: Tumor control dose 50%
TCGA: The cancer genome atlas
UHPLC: Ultra-high-performance liquid chromatography system
XBP1: X-box binding protein 1
