# Supplementary material for: Dual role of ER stress in response to metabolic co-targeting and radiosensitivity in head and neck cancer cells
Source: Cell Mol Life Sci. 2020 Nov 23;78(6):3021–44. doi: 10.1007/s00018-020-03704-7 (PMC8004506; doi:10.1007/s00018-020-03704-7)
Supplement: Supplementary file 1 — Supplementary file1 (PDF 751 KB) [file 18_2020_3704_MOESM1_ESM.pdf]

Table S1. Primers used for the detection and quantification of ER stress genes by RT- and q-PCR.

Target gene	Primer sequence	Product size
ACTB	Forward - CACCCTGAAGTACCCCATCG	199 bp
	Reverse - GCTGGGGTGTTGAAGGTCTC	
ATF3	Forward - GTGAATGCTGAACTGAAGGC	193 bp
	Reverse - CCATACCACGACTGCTTAGC	
ATF4	Forward - GGTCAGTCCCTCCAACAACAG	182 bp
	Reverse - GACTAGGGGGGCAAAGAGATCAC	
ATF6	Forward - CACTGATGAGCTGCAATTGG	224 bp
	Reverse - GAGTTGAAGAATAAGAGTCC	
ATG12	Forward - GTGAATCAGTCCTTTGCTCC	242 bp
	Reverse - CATAGAGTAGACACATACTAAATAGATCAC	
BAX	Forward - CTGACGGCAACTTCAACTGG	162 bp
	Reverse - GTCTTGGATCCAGCCCAACAG	
BCL2	Forward - AGGACCTCGCCGCTGCAGAC	256 bp
	Reverse - ACTCAAAGAAGGCCACAATCC	
BCL2L11/BIM	Forward - CCTTCTGATGTAAGTTCTGAGTG	109 bp
	Reverse - GTGGCTCTGTCTGTAGGGA	
BID	Forward - GCAGCTCAGGAACACCAGCC	238 bp
	Reverse - GGCTAAGCTCCTCACGTAG	
CHOP/DDIT3	Forward - GAGATGGCAGCTGAGTCATTGC	239 bp
	Reverse - CTCTGGGAGGTGCTTGTGAC	
ASS1	Forward - GAGCTCTTCATGTACCTGAACG	196 bp
	Reverse - CCAGGCCTTGTTTGATTTTGC	
MYC	Forward - GGATTCTCTGCTCTCCTCGAC	220 bp
	Reverse - GCTGTGAGGAGGTTTGCTG	
GADD34/PPP1R15A	Forward - GAGACAGAGGAAGAGGAAGCT	235 bp
	Reverse - GGAAATGGACAGTGACCTTCTC	
HSP5A/GRP78	Forward - CATAAACCCAGATGAAGCTG	195 bp
	Reverse - GAAAAGATCTGAGACTTCTTGG	
ERN1/IRE1	Forward - CAGAATTGGTGCAGGCATCCC	203 bp
	Reverse - GGTGTCGTACATGGTGATGGTG	
SQSTM1/p62	Forward - GGAAGGTGAAACACGGACAC	229 bp
	Reverse - CCGTGCTCCACATCGATATCAAC	
ASL	Forward - GAAGCGGATCAATGTCCTGC	502 bp
	Reverse - CTCTTGGTGAATCTGCAGCG	
XBP1*	Forward - CCTTGTAGTTGAGAACCAGG	442 bp
	Reverse - GGGGCTTGGTATATATGTGG	416 bp

*primers for unspliced and spliced forms of the *XBP1* gene were according to Yoshida et al., 2001 [23]

Table S2. List of siRNA oligonucleotides used in the study for gene knockdown

siRNA	Sequences
**scrambled #1**	AGGUAGUGUAAUCGCCUUGTT
**scrambled #2**	UGCGCUAGGCCUCGGUUGCTT
**scrambled #3**	GCAGCUAUAUGAAUGUUGU
**IRE1 #1**	CGCUGCUGCUGCCCGGCCU
**IRE1 #2**	GAUGUCCCACUUUGUGUCC
**IRE1 #3**	GCGUCUUUUACUACGUAAU
**GADD34 #1**	GCUAGGACUCCUCUGGCAA
**GADD34 #2**	GGACACUGCAAGGUUCUGA
**GADD34 #3**	GGACAGUGAUACAGGAUCA
**ATF3 #1**	CAUUUGAUAUACAUGCUCA
**ATF3 #2**	GAAACCUCUUUAUCCAACA
**ATF3 #3**	GAAGGAACAUUGCAGAGCU
**ATF4 #1**	ACUUCAAACCUCAUGGGUU
**ATF4 #2**	CCACGUUGGAUGACACUUG
**CHOP #1**	CUGAUUGACGGAAUGGUGA
**CHOP #2**	GAACCAGCAGAGGUCACAA

Table S3. List of antibodies used in the study

Antibody (Ab)	Source	Cat. Number
Rabbit monoclonal anti-ATF4 Ab	Cell Signaling Technology	Cat# 11815; RRID: AB_2616025
Mouse monoclonal anti-AKT	Cell Signaling Technology	Cat# 2920S; RRID: AB_1147620
Rabbit monoclonal anti-Phospho-AKT (Ser475) Ab	Cell Signaling Technology	Cat# 4060S; PRID: AB_2315049
Rabbit monoclonal anti-Phospho-p44/42 MAPK (Erk1/2) (Thr202/Tyr204) Ab	Cell Signaling Technology	Cat# 4370; RRID: AB_2315112
Rabbit polyclonal anti-p44/42 MAPK (Erk1/2) Ab	Cell Signaling Technology	Cat# 9102S; RRID: AB_330744
Rabbit monoclonal anti-Phospho-eIF2alpha (Ser51) Ab	Cell Signaling Technology	Cat# 3597S; RRID: AB_390740
Rabbit monoclonal anti- eIF2alpha Ab	Cell Signaling Technology	Cat# 5324; RRID: AB_10692650
Rabbit monoclonal anti-cleaved PARP (Asp214) Ab	Cell Signaling Technology	Cat# 5625S; RRID: AB_10699459
Rabbit polyclonal anti-LC3B Ab	Cell Signaling Technology	Cat# 4108; RRID: AB_2137703
Rabbit monoclonal anti-Bim Ab	Cell Signaling Technology	Cat# 2933; RRID: AB_1030947
Rabbit monoclonal anti-Phospho-p70 S6 Kinase (Thr389) Ab	Cell Signaling Technology	Cat# 9234S; RRID: AB_2269803
Monoclonal anti-p70 S6 Kinase Ab	Cell Signaling Technology	Cat# 2708; RRID: AB_390722
Rabbit polyclonal anti-ASS1 Ab	Sigma-Aldrich	Cat# HPA020934; RRID: AB_1845118
Mouse monoclonal anti-Tubulin(alpha) Ab	Millipore	Cat# 05-829; RRID: AB_310035
Rabbit polyclonal anti-GAPDH Ab	Santa Cruz Biotechnology	Cat# sc-25778; RRID: AB_10167668

Table S4A Statistical significance of 2-D HNSCC growth and regrowth capacity upon mono-ADT

Significance levels (p values) for differences in overall growth and regrowth of HNSCC cell lines exposed to various treatment conditions for increasing time intervals are documented. Treatment groups of interest were compared for all time points using a two ways ANOVA test; p-values <0.05 were considered as statistically significant.

Condition / Cell line	–Arg *vs.* +Arg	–Arg (+3d recovery) *vs.* –Arg	–Arg +Cit *vs.* +Arg	–Arg +Cit (+3d recovery) *vs.* –Arg +Cit
**SAS**	<0.001	n.s.	<0.001	<0.001
**HSC4**	<0.001	n.s.	<0.001	<0.001
**Cal-33**	<0.001	<0.001	<0.001	<0.001
**XF345**	<0.001	n.s.	<0.001	<0.001
**FaDu**	<0.001	<0.001	<0.001	<0.001
**UT-SCC-5**	<0.001	<0.001	<0.001	<0.001
**UT-SCC-8**	<0.001	<0.01	<0.001	<0.001
**UT-SCC-14**	<0.001	n.s.	<0.001	<0.001
**UT-SCC-15**	<0.001	<0.001	<0.001	<0.001

+Arg, arginine-rich medium; –Arg, arginine-free medium; +Cit, 0.04 mM citrulline supplement
n.s., not statistically significant

Table S4B Statistical significance of surviving fraction dose response curves of HNSCC with and without mono-ADT

Significance levels (p values) for differences in clonogenic survival after single dose irradiation in the absence and presence of Arginine (Arg) and Citrulline (Cit), respectively, are given for the HNSCC cell line panel. Cell lines are listed in alphabetical order. The statistical procedure applied in IBM SPSS Statistics 25.0 is described in *Materials and Methods*.

Condition / Cell line	+Arg +Cit *vs.* +Arg	–Arg *vs.* +Arg	–Arg +Cit *vs.* +Arg	–Arg +Cit *vs.* –Arg	Radio-sensitization by mono-ADT	Impact of Cit on mono-ADT-induced radio-sensitization
**SAS**	n.s.	<0.001	<0.001	n.s.	yes	n.s.
**HSC4**	n.s	<0.001	<0.01	n.s.	yes	n.s.
**Cal-33**	n.s.	<0.001	<0.001	n.s.	yes	n.s.
**XF345**	n.s.	<0.001	<0.01	<0.05	yes	reduction
**FaDu**	n.s.	n.s.	n.s.	n.s.	no	-
**UT-SCC-5**	n.s.	<0.001	<0.001	<0.01	yes	reduction
**UT-SCC-8**	n.s.	<0.05	<0.01	n.s.	yes	n.s.
**UT-SCC-14**	n.s.	n.s.*	<0.05	n.s.	tendency	n.s.
**UT-SCC-15**	n.s.	n.s.	n.s.	n.s.	no	-

+Arg, arginine-rich medium; –Arg, arginine-free medium; +Cit, 0.04 mM citrulline supplement
n.s., not statistically significant; *tendency (p= 0.053)

Table S5A Statistical significance of SAS and FaDu 2-D growth and regrowth capacity upon comb-ADT

The calculation of statistical significance was performed according to Table S4A.

**Condition 1** *vs.*	**Condition 2**	**SAS**	**FaDu**
+Arg	+Arg + 0.05 mM Cav	n.s.	n.s.
+Arg	+Arg + 0.10 mM Cav	n.s.	n.s.
–Arg	–Arg + 0.05 mM Cav	<0.001	<0.001
–Arg	–Arg + 0.10 mM Cav	<0.001	<0.001
–Arg + 0.05 mM Cav	–Arg + 0.1 mM Cav	n.s.	n.s.
–Arg (+3d recovery)	–Arg + 0.05 mM Cav (+3d recovery)	<0.001	<0.001
–Arg (+ 3d recovery)	–Arg + 0.10 mM Cav (+ 3d recovery)	<0.001	<0.001
–Arg + 0.05 mM Cav (+ 3d recovery)	–Arg + 0.10 mM Cav (+ 3d recovery)	n.s.	n.s.

+Arg, arginine-rich medium; –Arg, arginine-free medium; +Cav, 0.05-0.1 mM canavanine;
n.s., not statistically significant

Table S5B Statistical significance of surviving fraction dose response curves of SAS and FaDu cells with and without comb-ADT

The calculation of statistical significance was performed according to Table S4B.

**Condition 1** *vs.*	**Condition 2**	**SAS**	**FaDu**
+Arg	+Arg + 0.01 mM Cav	n.s.	n.d.
+Arg	+Arg + 0.05 mM Cav	n.s.	n.s.
+Arg	+Arg + 0.10 mM Cav	n.d.	n.s.
+Arg+ 0.01 mM Cav	+Arg+ 0.05 mM Cav	n.s.	n.d.
+Arg+ 0.05 mM Cav	+Arg+ 0.10 mM Cav	n.d.	n.s.
+Arg	–Arg +Cit	<0.001	n.s.
+Arg	–Arg +Cit + 0.01 mM Cav	<0.001	n.d.
+Arg	–Arg +Cit + 0.05 mM Cav	<0.001	<0.001
+Arg	–Arg +Cit + 0.10 mM Cav	n.d.	<0.001
–Arg	–Arg +Cit + 0.01 mM Cav	n.s.	n.d.
–Arg	–Arg +Cit + 0.05 mM Cav	<0.01	<0.001
–Arg	–Arg +Cit + 0.10 mM Cav	n.d.	<0.001
–Arg + 0.01 mM Cav	–Arg +Cit + 0.05 mM Cav	n.s.	n.d.
–Arg + 0.05 mM Cav	–Arg +Cit + 0.10 mM Cav	n.d.	n.s.

+Arg, arginine-rich medium; –Arg, arginine-free medium; +Cit, 0.04 mM citrulline supplement
n.d. not determined; +Cav, 0.01-0.1 mM canavanine; n.s., not statistically significant

Table S6A Statistical significance of surviving fraction dose response curves of SAS cells under mono-ADT upon (pre)-expose to ER stress modulators

The calculation of statistical significance was performed according to Table S4B.

**Condition 1** *vs.*	**Condition 2**	**SAS**	Impact on mono-ADT-induced radiosensitization
+Arg	+Arg +DMSO	n.s.	n.a.
+Arg	+Arg +Sal	n.s.	n.a.
+Arg	–Arg	<0.001	n.a.
+Arg	–Arg +DMSO	<0.001	no full protection
+Arg	–Arg +Sal	<0.001	no full protection
–Arg	–Arg +DMSO	<0.001	reduced efficacy
–Arg	–Arg +Sal	<0.001	reduced efficacy

+Arg, arginine-rich medium; –Arg, arginine-free medium; 2% DMSO; 0.02 mM Salubrinal (Sal)
n.s., not statistically significant; n.a., not applicable

Table S6B Statistical significance of surviving fraction dose response curves in SAS cells with and without ER stress gene knockdown

**Condition 1** *vs.*	**Condition 2**	**SAS**	Impact on radioresponse
+Arg siScr	+Arg siIRE1	<0.01	minor sensitization
+Arg siScr	+Arg siCHOP	n.s.	none
+Arg siScr	+Arg siATF3	n.s.	none
+Arg siScr	+Arg siATF4	<0.001	sensitization
+Arg siScr	+Arg siGADD34	n.s.	none
+Arg siScr	+Arg siGADD34 + siATF4	<0.001	sensitization
+Arg siScr	–Arg siScr	<0.001	sensitization
+Arg siScr	–Arg siIRE1	<0.001	sensitization
+Arg siScr	–Arg siCHOP	<0.001	sensitization
+Arg siScr	–Arg siATF3	<0.001	sensitization
+Arg siScr	–Arg siATF4	<0.001	sensitization
+Arg siScr	–Arg siGADD34	<0.001	sensitization
+Arg siScr	–Arg siGADD34 + siATF4	<0.001	sensitization
–Arg siScr	–Arg siIRE1	n.s.	none
–Arg siScr	–Arg siCHOP	<0.05	minor sensitization
–Arg siScr	–Arg siATF3	<0.01	minor protection
–Arg siScr	–Arg siATF4	n.s.	none
–Arg siScr	–Arg siGADD34	<0.05	minor protection
–Arg siScr	–Arg siGADD34 + siATF4	n.s.	(none/minor protection)

+Arg, arginine-rich medium; –Arg, arginine-free medium; n.s., not statistically significant

Table S7A SCD$_{50}$ for SAS and FaDu spheroids, which were exposed to different treatment conditions before irradiation

Condition	SAS		FaDu	
	**SCD$_{50}$, Gy**	**DRF [95% CI]**	**SCD$_{50}$, Gy**	**DRF [95% CI]**
+Arg (control)	18.8	–	13.0	–
+ Arg + Cav	19.2	0.98 [0.93-1.03]	13.3	0.97 [0.91-1.04]
– Arg	11.5	1.63* [1.54-1.74]	10.1	1.28* [1.19-1.37]
– Arg + Cit	11.6	1.59* [1.51-1.68]	11.9	1.09* [1.02-1.19]
– Arg + Cit + Cav	9.6	1.94* [1.86-2.10]	9.3	1.40* [1.30-1.49]

+Arg, arginine-rich medium; –Arg, arginine-free medium; +Cit, 0.04 mM citrulline supplement; Cav, 0.10 mM canavanine; SCD$_{50}$, spheroid control dose 50; DRF, dose reduction factor with 95% confidence interval (CI) relative to SCD$_{50}$ in arginine-rich medium (control)

Table S7B Statistical significance of SCD$_{50}$ values

Significance levels (p values) for differences of SCD$_{50}$ values from SAS (grey layout) and FaDu spheroids, respectively, exposed to the various treatment conditions. The specific bootstrapping procedure and software applied for this statistical analysis is described in Materials and Methods.

FaDu \ SAS	+Arg (control)	+Arg +Cav	–Arg	–Arg +Cit	–Arg +Cit +Cav
+Arg (control)		n.s.	<0.001	<0.001	<0.001
+Arg +Cav	n.s		---	---	---
–Arg	<0.001	---		n.s.	<0.001
–Arg +Cit	<0.05	---	<0.001		<0.001
–Arg +Cit +Cav	<0.001	---	<0.05	<0.001	

+Arg, arginine-rich medium; –Arg, arginine-free medium;
+Cit, 0.04 mM citrulline supplement; Cav, 0.10 mM canavanine;
overlapping treatment conditions are marked in black;
white background – FaDu, grey background - SAS
