# Supplementary material for: Dual role of ER stress in response to metabolic co-targeting and radiosensitivity in head and neck cancer cells
Source: Cell Mol Life Sci. 2020 Nov 23;78(6):3021–44. doi: 10.1007/s00018-020-03704-7 (PMC8004506; doi:10.1007/s00018-020-03704-7)
Supplement: Supplementary file 2 — Supplementary file2 (PDF 1345 KB) [file 18_2020_3704_MOESM2_ESM.pdf]

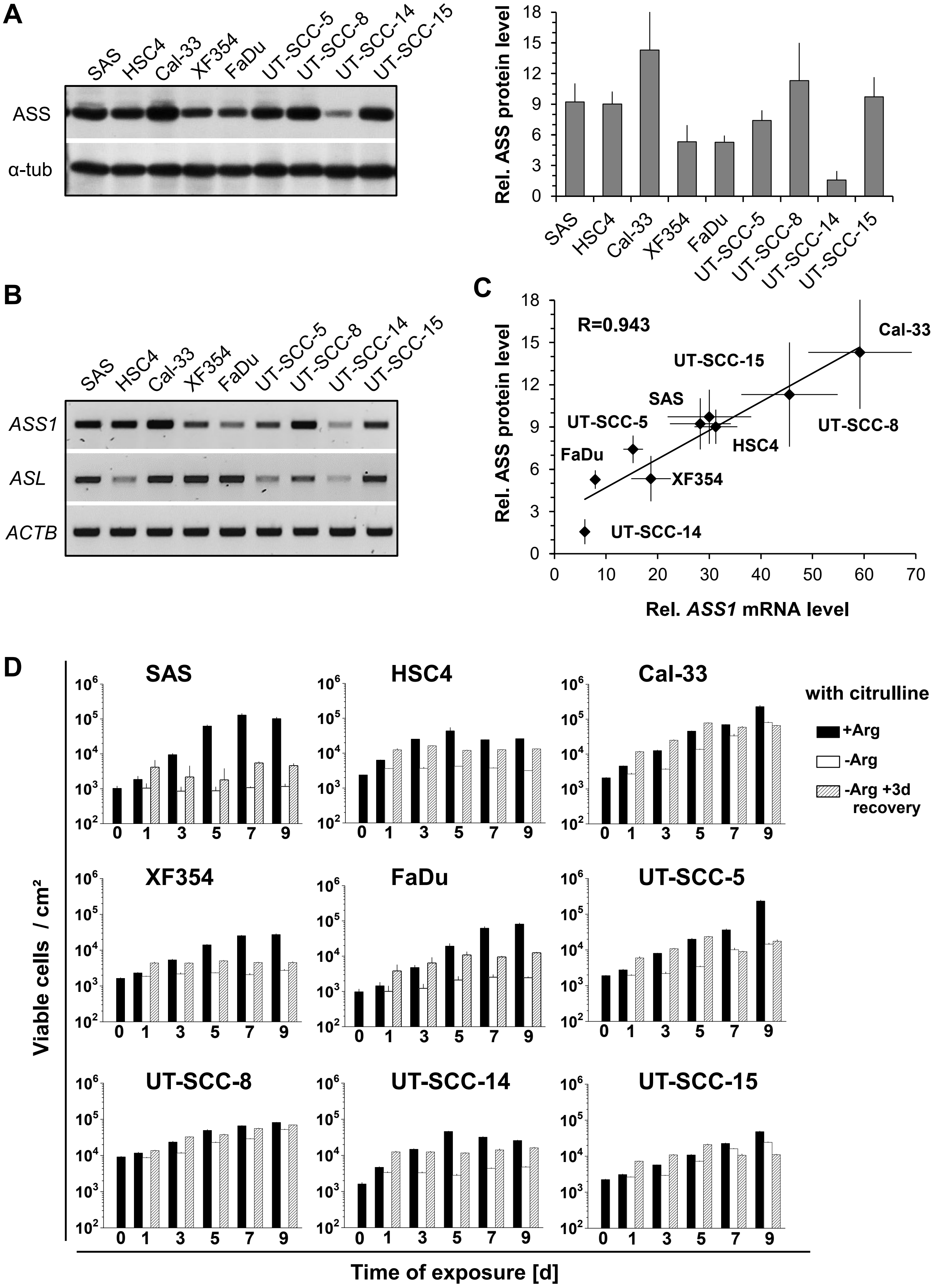

Figure S1 – see legend on next page

Figure S1

Heterogeneous ASS1 expression pattern in HNSCC cell lines does not correlate with their potential for compensating ADT-induced growth arrest and loss of regrowth capacity in the presence of citrulline (Cit).

(A) Representative Western blot and semi-quantitative densitometric analysis of relative intrinstic ASS protein level in the HNSCC cell line panel when grown as exponential monolayer culture under standard conditions. α-tubulin (α-tub) was used as loading control. Data represent means +SD of N=3 independent experiments.

(B) Representative RT-PCR analysis of *ASS1 and ASL* genes in HNSCC cell lines grown according to (A). *ACTB* mRNA level was recorded as reference.

(C) Correlation analysis of the expression of ASS1 gene and protein levels in the HNSCC cell lines determined by RT-PCR and Western blot analysis.

(D) Growth behavior of HNSCC cells under mono-ADT in the presence of Cit. Cells were exposed to Arg-free medium (-Arg) containing physiological Cit concentrations (0.04 mM) for up to 9 days. Selected samples were assessed for growth recovery by exchanging the supernatant to standard complete medium (+Arg) after 1d, 3d, 5d, 7d or 9d of exposure to the Arg-deprived condition; cell counts were measured 3 days later. Graphs show mean values +SD from N=3 independent experiments.

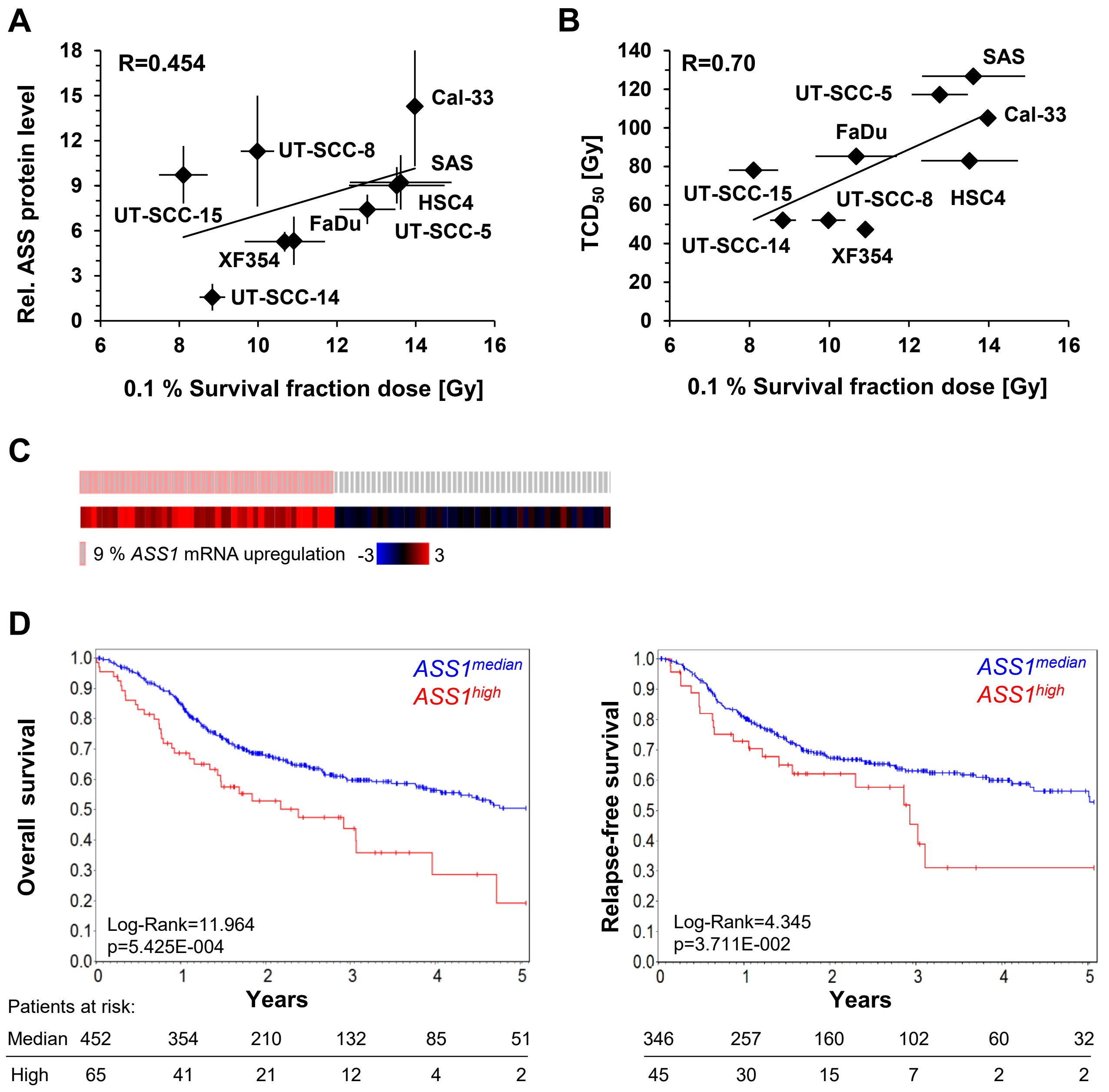

Figure S2

High level of *ASS1* expression is a marker of poor prognosis for HNSCC patients and radioresponse.

(A) Correlation analysis of the expression of *ASS1* gene and 0.1% surviving fraction (SF) doses of HNSCC lines determined *in vitro*.

(B) Correlation analysis of 0.1% SF doses determined *in vitro* and TCD_{50} (dose required to control the disease in 50% of tumor-bearing animals) of the corresponding xenograft models of HNSCC lines published previously [20].

(C) Oncoprint of human HNSCC with upregulated expression of *ASS1* gene based on the data in The Cancer Genome Atlas (TCGA; 517 non-randomized tumors; 1.5 z-score).

(D) Kaplan–Meier analysis to assess the correlation of *ASS1* expression with overall and relapse-free survival of HNSCC patients from the TCGA (517 non-randomized tumors) database. Data were stratified by *ASS1* expression level: *ASS1*^{median} and *ASS1*^{high} (≥3 f.c upregulation; correspondent 1.5 z-score) and comparative analysis by log-rank test was performed between these two different groups.

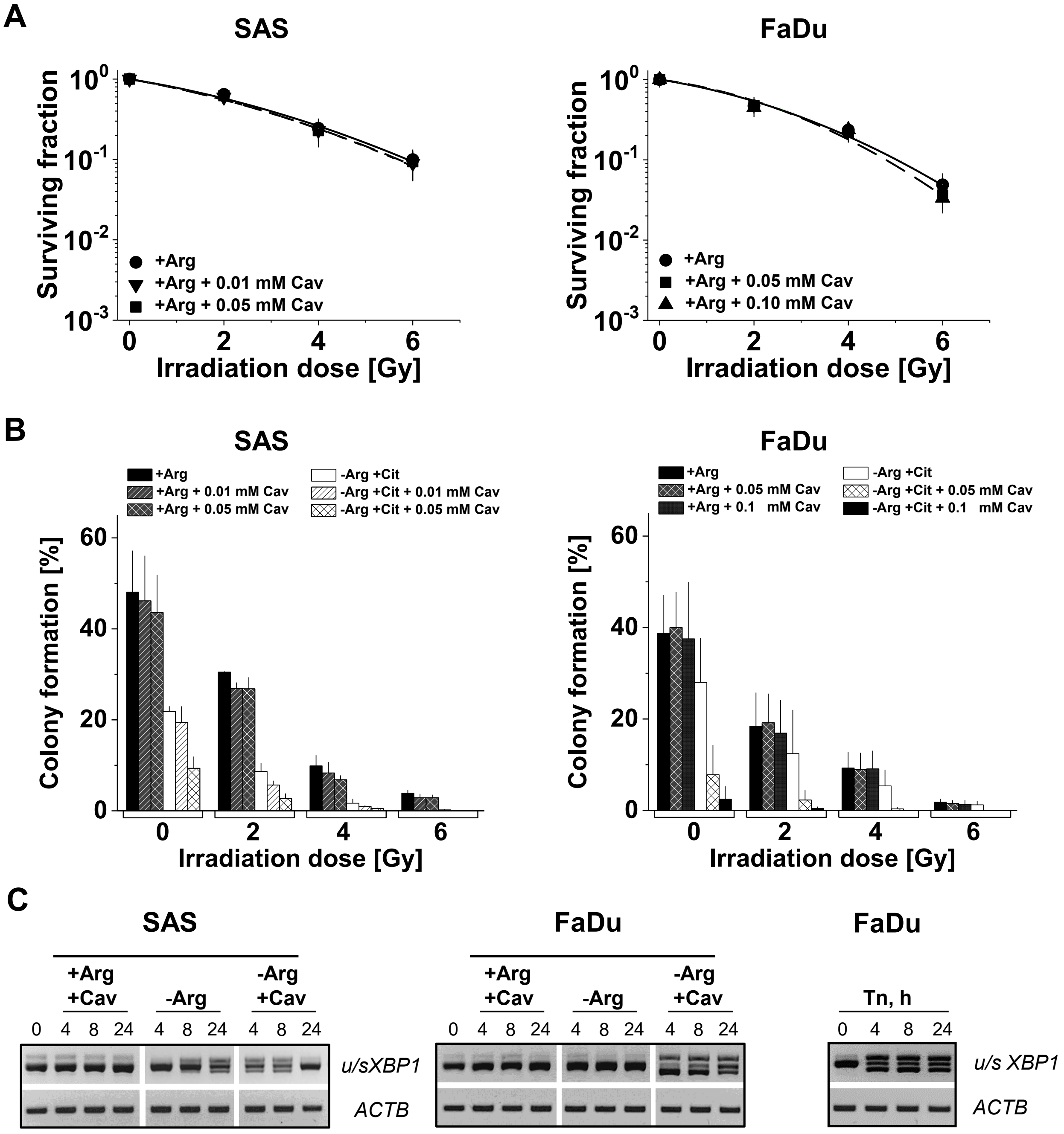

Figure S3

Cav at low concentrations is cytotoxic for HNSCC cells only in an Arg-free environment.

(A) Surviving fractions (±SD) of SAS and FaDu cells irradiated under standard culture conditions (+Arg) in the absence or presence of 0.04 mM Cit and 0.01-0.1 mM Cav (N=3, n≥3). Data were fitted with a linear-quadratic model as depicted in Materials and Methods.

(B) Colony forming capacity of SAS and FaDu cells treated by comb-ADT with 0.04 mM Cit and 0.05-0.1 mM Cav upon 0-6 Gy single dose irradiation.

(C) Representative RT-PCR analyses of spliced *XBP1* expression in monolayer SAS and FaDu cells after defined times of Cav (0.1 mM) exposure. The spliced *XBP1* mRNA (lower band) is apparent already after 4 h of comb-ADT; *ACTB* is shown as reference control and FaDu cells treated with tunicamycin (Tn; 3 µg/ml) served as positive control (right).

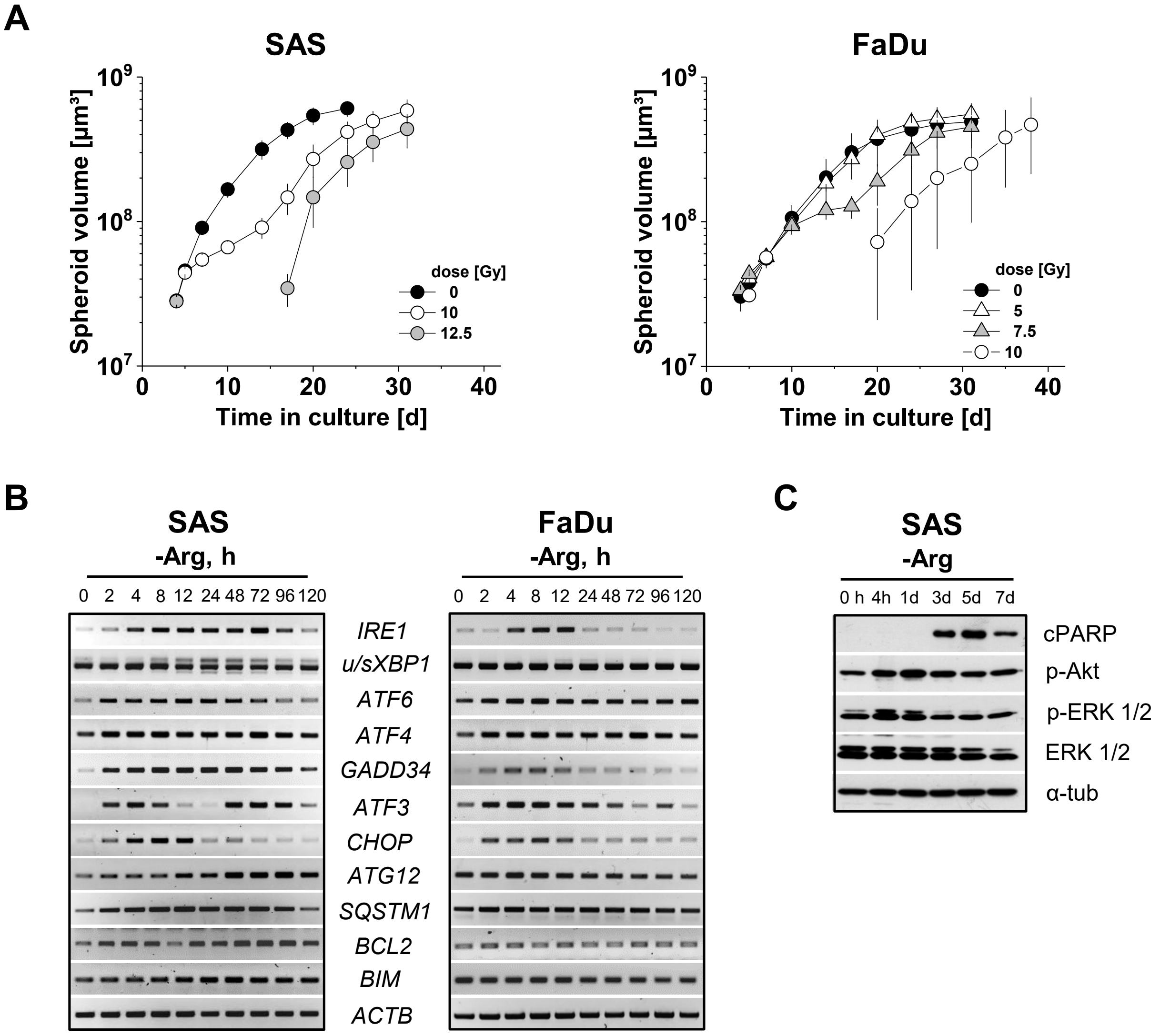

Figure S4

Single dose irradiation alone leads to spheroid growth delay and control; mono-ADT less effectively triggers ER stress in the 3-D environment than in 2-D culture (cf. Figs. 6 and 2).

(A) Spheroid volume growth is delayed after irradiation of 3-D cultures with single doses that do not yet abrogate growth recovery (up to 12.5 Gy for SAS and 10 Gy for FaDu spheroids); mean values (± SD) are shown for n≥28 spheroids.

(B) Representative data sets of ER stress response genes in SAS and FaDu spheroids analyzed by RT-PCR. Spheroids were incubated in Arg-free medium for up to 120 h or left untreated (0 h). *ACTB* mRNA level was determined as reference.

(C) Representative Western blots of proteins from SAS spheroids upon mono-ADT. Cell lysates were probed with specific antibodies against the indicated proteins; α-tubulin (α-tub) was used as loading control.

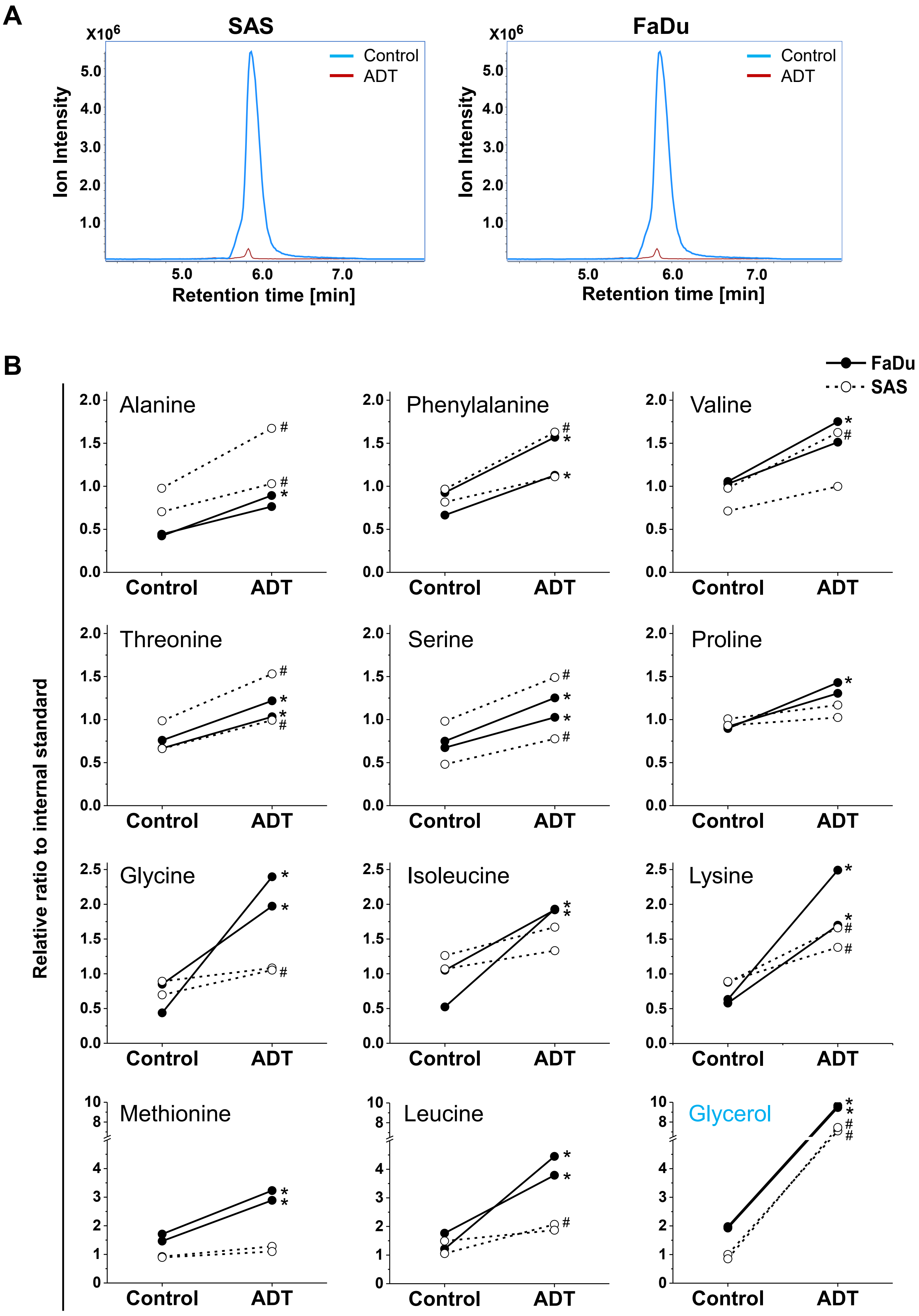

Figure S5 – see legend on next page

C

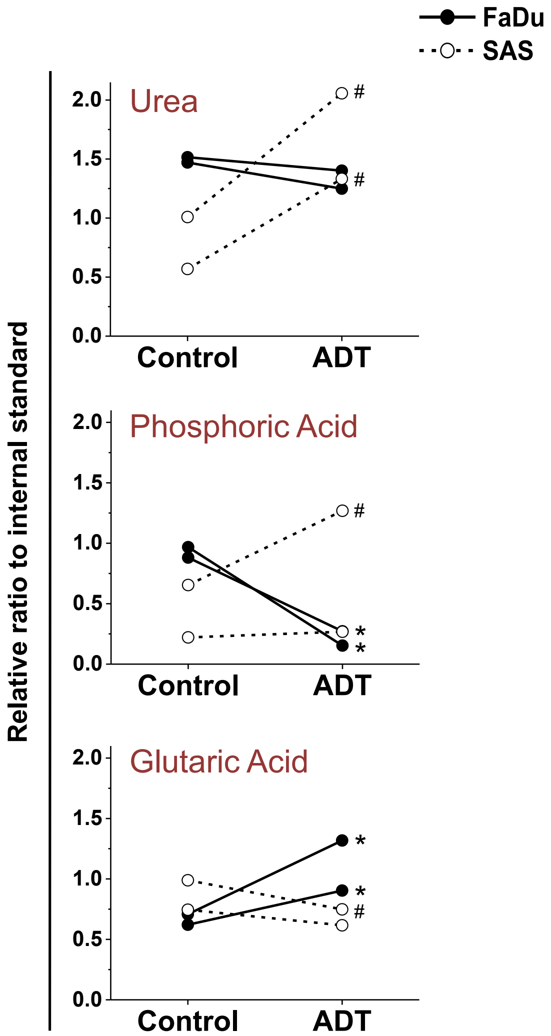

Figure S5

Mono-ADT induces specific metabolic alterations in HNSCC spheroids related to protein degradation and amino acid metabolism

(A) Intracellular arginine level detected via LC-MS in SAS and FaDu spheroids under control and mono-ADT conditions

(B) Effect of mono-ADT on intracellular levels of certain similarly regulated molecules (amino acids and glycerol) in SAS and FaDu spheroids

(C) Effect of mono-ADT on intracellular levels of certain differently regulated metabolites in SAS and FaDu spheroids

Data in **(B)** and **(C)** show the means of two independent experiments (n=3-6 technical repeats) measured using GCxGC-qMS (fold change ≥1.5; log2 value treated vs. untreated ≥0.584; *p≤0.01 significant in FaDu spheroids; # p≤0.01 significant in SAS spheroids

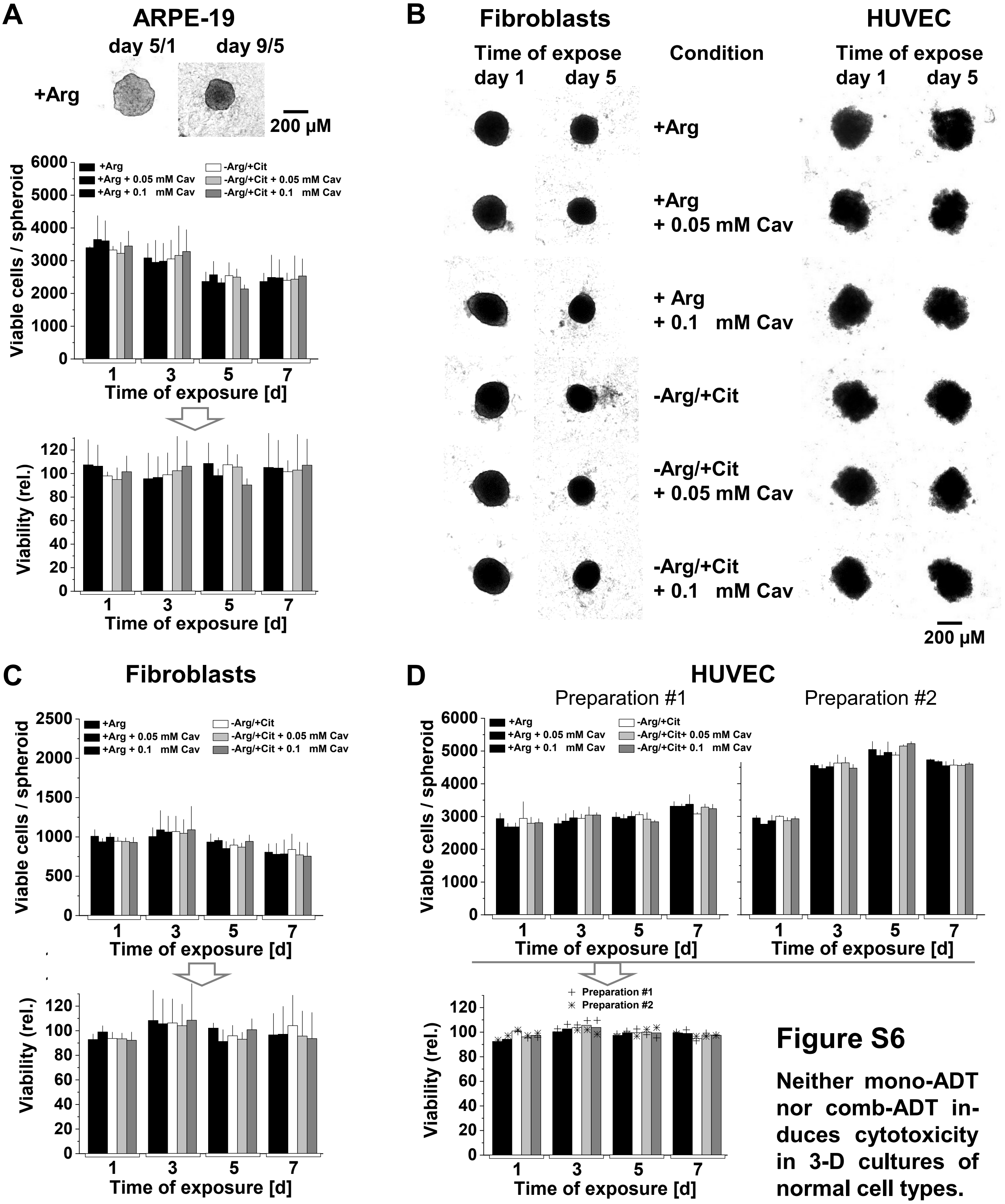

Figure S6

Neither mono-ADT nor comb-ADT induces cytotoxicity in 3-D cultures of normal cell types.

(**A**) (from top to bottom) Representative images of discoidal aggregates of retinal pigment epithelial cells under control conditions (+Arg) at days 5 and 9 in culture (≙ days 1 and 5 of exposure to treatment); mean number of membrane-intact ARPE-19 cells per “spheroid” (±SD) and the respective cell viabilities upon treatment relative to the corresponding (+Arg) control cultures (N≥3).

(B) Representative phase contrast images of fibroblast and HUVEC spheroid cultures exposed to various treatment conditions for 1 and 5 days.

(C) Mean number of membrane-intact fibroblasts per spheroid and relative cell viabilities (±SD) according to (A) (N≥3)

(D) Number of membrane-intact HUVEC per spheroid with intraexperimental variation (N=1, n=3 biological repeats) for two different HUVEC preparations and relative cell viabilities upon treatment (related to corresponding control cultures of the same preparation and day of exposure)
